# Root restriction accelerates genomic target identification in quinoa under controlled conditions

**DOI:** 10.1111/ppl.70223

**Published:** 2025-04-15

**Authors:** Davide Visintainer, Nanna Fjord Sørensen, Mengming Chen, Mai Duy Luu Trinh, Rute R. da Fonseca, Sara Fondevilla, Rosa L. López‐Marqués

**Affiliations:** ^1^ Copenhagen Plant Science Center, Department of Plant and Environmental Sciences University of Copenhagen Frederiksberg Denmark; ^2^ Center for Global Mountain Biodiversity, Globe Institute University of Copenhagen Copenhagen Denmark; ^3^ Instituto de Agricultura Sostenible, Avenida Menéndez Pidal s/n, Alameda del Obispo Campus Consejo Superior de Investigaciones Científicas (CSIC) Córdoba Spain

## Abstract

Quinoa (*Chenopodium quinoa*) is a nutritious and resilient crop that displays a high genetic and phenotypic variation. As the popularity of this crop increases, there is a growing need to integrate classic and modern breeding tools to favor its improvement. We tested root restriction as a method to reduce plant size and enable high‐throughput phenotypic screening of large sets of quinoa plants under controlled conditions. We verified how increasing root restriction does not affect the prediction of field behavior with respect to other standard greenhouse cultivation procedures. We then combined the phenotypic information obtained with our root restriction system with whole‐genome re‐sequencing data to characterize a quinoa diversity panel of 100 accessions and showed that phenotypic data obtained from root‐restricted plants provide real insights into quinoa genetics. Finally, we carried out a genome‐wide association study (GWAS) and identified a previously described locus for betalain biosynthesis, as well as other candidate loci linked to betalain biosynthesis and seed size. Overall, we showed that a phenotyping system based on root restriction can aid the identification of genomic targets in quinoa, which can complement and inform field trials for certain traits. This work supports further breeding and faster improvement of quinoa.

## 1 INTRODUCTION

Domestication and improvement of orphan crops are currently explored as an option to enrich and diversify the agricultural arsenal and to create longer and tailored crop rotations (Stetter et al., 2017). One such crop, quinoa (*Chenopodium quinoa*), was partially domesticated in South America around 7,000 years ago. It displays a rich genetic and phenotypic diversity, which reflects its diffusion across drastically different agroecological environments (Christensen et al., 2007; Fuentes et al., 2009; Mason et al., 2005; Ruas et al., 1999). Quinoa is tolerant to a wide array of environmental stresses, including salinity and drought, and presents an exceptional nutritional profile (Ruiz et al., 2014).

Considering its agricultural potential, various quinoa breeding programs were initiated in Bolivia, Peru, Ecuador, and Chile in the 1960s. Despite these efforts, quinoa can still be considered a partially domesticated crop, with yields reported to oscillate between 1–3 tons per hectare year (t/ha), depending on the setting and cultivation practices (Aguilar & Jacobsen, 2003; Pulvento et al., 2012; Rezzouk et al., 2020; Shah et al., 2020). This is still very low compared to established crops like wheat, which can reach 7–11 t/ha (Asseng et al., 2015; Bayliss‐Smith & Wanmali, 1984).

To bring quinoa to the level of other conventional staples, it is necessary to integrate modern tools into the breeding process. Efforts in this direction have been initiated only recently. The first chromosome‐level reference genome of quinoa was available in 2017 (Jarvis et al., 2017) and the first phenotyping standardized guidelines were discussed between 2017 and 2022 (Sosa‐Zuniga et al., 2017; Stanschewski et al., 2021). The availability of these resources led to the first studies aimed at dissecting the genetic basis of quinoa agronomic traits in biparental crossing populations and diversity panels (Colque‐Little et al., 2021; Fondevilla et al., 2024; Maldonado‐Taipe et al., 2022; Mizuno et al., 2020; Nepal et al., 2023; Patiranage et al., 2022). However, despite the expansion of extensive collaboration networks (Murphy et al., 2016), studies that explore the repertoire of plant genetic diversity are extremely labor‐intensive. Indeed, while modern approaches such as remote sensing are being deployed to facilitate quinoa phenotyping in the field (Jaramillo Roman et al., 2021; Jiang et al., 2022), high throughput techniques generating phenotyping data are under constant demand (Furbank & Tester, 2011; Mir et al., 2019).

To speed up the process of quinoa improvement, we sought to evaluate the benefits of growing and screening quinoa plants under controlled conditions, making use of root restriction. Root restriction is a way to inhibit plant growth by raising plants in small containers. This method has been widely employed in the model plant *Arabidopsis thaliana* and in horticulture and vertical farming (*Eurasian Arabidopsis Stock Centre* (uNASC), 2023; Graham & Wheeler, 2016; van Iersel, 1997; NeSmith et al., 1992; Zhu et al., 2006).

In our experimental framework, controlled conditions provide a standardized platform for phenotyping, also allowing to conduct year‐round research (Wang et al., 2021). The combination of controlled conditions with root restriction, which dramatically reduces the amount of space required, could potentially alleviate both the repeatability and the scalability problem of screening studies. Indeed, some studies have already used controlled conditions with various degrees of root restriction (Patiranage et al., 2021; Tovar et al., 2020). However, while they focus on specific traits and environmental parameters, our work aims to demonstrate the reliability of root restriction as a method for high‐throughput phenotyping under controlled conditions. We achieve this by 1) comparing agronomical traits under controlled root‐restricted versus field conditions, 2) using the setup to characterize a diversity panel comprised of 100 quinoa accessions and carrying out a genome‐wide association study (GWAS) focused on 6 traits of agronomic relevance.

## 2 MATERIALS AND METHODS

### 2.1 Plant material

All *Chenopodium quinoa* plant germplasm from South America was obtained through IPK Gatersleben (https://www.ipk-gatersleben.de/). Titicaca and Vikinga are the standard accessions routinely used in our laboratory, and seeds were originally donated by Prof. Sven‐Erik Jacobsen (Quinoa Quality ApS, Denmark). In order to increase the homogeneity of the accessions, we performed one and two rounds of selfing in plants obtained by single seed descent from the original germplasm library for the root restriction experiment and the GWAS, respectively.

### 2.2 Growing conditions

Seeds for plants grown in the greenhouse were surface‐sterilized by soaking them in a bleaching solution containing 0.5% (w/v) calcium hypochlorite [Ca(ClO)₂] and 0.001% (v/v) Tween‐20 overnight at 4°C and sown the next day.The temperature was kept between 18–25°C during the day and 16–20°C during the night. For plants grown during the summer, the temperature rose often above this threshold, as aeration alone could not compensate for the greenhouse effect. The substrate used was a peat standard mixture with slow‐release fertilizer (Grona Linien, Urnjord). Plants were irrigated with fertilizer until flowering. Afterwards, normal tap water was used. Humidity was kept between 60–70%.

Restricted plants were sown in Aratrays (Arasystems 28 wells, volume 100 mL) in 8 replicates per accession. Standard plants were sown in 2‐liter containers with 4 replicates per accession. Replicate samples were randomly distributed within the greenhouse to account for possible variability.

For the root restriction experiment, artificial light was provided by sodium lamps, yielding between 100–200 μmol m^−2^ s‐1). Plants were irradiated for 16 hours a day (long day) to reflect field growing conditions at high latitudes.

For plants used in the GWAS, artificial light was provided by full spectrum Horti LED lamps (Horti Lux) yielding 200–300 μmol m^−2^ s^−1^, while darkness was ensured by automatic curtains.

For the open field setting, plants were sown in a plot at the Experimental farm (University of Copenhagen, Denmark) in Taastrup (Plot identifier: field 19). The soil was fertilized 2 weeks prior, using organic fertilizer and the field was harrowed on the same day of sowing. A volume of 5 mL of dry seeds per accession was directly sown in 3‐m rows using a manual precision seeder at approximately 1–2 mm depth, without previous surface‐sterilization. Three weeks after germination, seedlings were trimmed down to 20–25 plants per accession, keeping a distance of 10 cm between plants and 50 cm between rows. Due to space limitations, each accession was present in 1 block, aside from the Danish line Titicaca, which was sown around the plot and in 6 replicate blocks to test for homogeneity across the field (Figure S1a). This served to account for environmental factors within the plot and to create a buffer zone for pests and soil from adjacent plots. The plot was irrigated only during germination. After maturity and senescence, full plants were harvested and dried in a barn before seed cleaning and scoring of post‐harvest traits. The specific lines used for each setting are reported in Table S1.

### 2.3 Scoring of agronomic traits

Scored traits were weeks to flowering, dry plant biomass, seed diameter, total weight of seeds per plant, hundred‐kernel‐weight and stem pigmentation. Flowering time was scored weekly or bi‐weekly according to the quinoa BBCH scale (Sosa‐Zuniga et al., 2017). Stem pigmentation was scored after flowering using a scale of 1–5 (1: no pigmentation, 2: faint traces of red, 3: clear interspersed red patches, 4: majority of stem purple, 5: stem completely purple) and excluding coloration of axils from the assessment. After harvesting and drying the plants, individual plants were weighed to measure the dry biomass. For each plant, seeds were harvested and cleaned separately using an air seed thrasher. Afterwards, 100–300 seeds were poured into flat containers with a blue‐colored bottom and placed on top of a scale (Ohaus Adventurer, max 3100 g, readability 0.01 g). Seeds were imaged using a smartphone placed on a fixed support and the weight was annotated. Using a custom‐made macro script in ImageJ, we counted the seeds for each image and determined the average diameter. The hundred‐kernel‐weight was calculated from the number of seeds in the picture and the weight recorded on the scale at the time of imaging (Figure S2). The total seed weight per plant was also recorded on the scale.

### 2.4 Genomic DNA extraction

Healthy leaf tissue samples were obtained from a first generation of selfed plants (S1) for whole genome sequencing. DNA was extracted using a version of the CTAB method, as explained previously (Trinh et al., 2024). The eluted DNA was gently resuspended in 50 μL of nuclease‐free water and stored at −20°C.

### 2.5 Genomic data processing

The data used for population structure and variant calling used in this study were generated in a previous work, where all data processing methods are also described (Trinh et al., 2024). Briefly, we used whole genome re‐sequencing data from 124 individual accessions, obtained with NovaSeq Illumina with 150 bp paired‐end reads, with 6X to 10X coverage. Of these, 34 raw reads datasets were downloaded from the NCBI Sequence Read Archive (SRA, https://www.ncbi.nlm.nih.gov/sra). The accession IDs and SRA identifiers are found in Table S2. Read filtering and trimming were performed using fastp (v0.23.4) (Chen et al., 2018). Reads were mapped to the V1 quinoa reference genome (CoGe ID: 33827) using minimap2 (v2.26) (H. Li, 2018). Read grouping and duplicate removal were performed with GATK4 AddOrReplaceReadGroups (Picard) and MarkDuplicates (Picard) tools (v4.4.0.0) (DePristo et al., 2011; McKenna et al., 2010). Genotype likelihoods were calculated using ANGSD (v0.940‐stable) (Korneliussen et al., 2014). We used PCAngsd (v1.2) (Meisner & Albrechtsen, 2018) to generate the eigenvectors and eigenvalues. Variant calling was performed with bcftools (v1.8) mpileup piped into bcftools call (Lefouili & Nam, 2022). Both bcftools and vcftools (v0.1.16) (Danecek et al., 2011) were used for variant filtering. For LD decay calculations, we used a SNP set obtained by using a MAF of 0.01 as filtering parameter on the main set, after imputation. We calculated the r^2^ correlation coefficient between SNPs using the PopLDdecay software (v3.42) with default settings (‐MaxDist 300) (C. Zhang et al., 2019). The estimated LD decay was determined by setting the threshold at r^2^ = 0.2. For calculation of LD plots in selected regions, we used the tool LDkit (v1.0) (Tang et al., 2020).

For admixture analysis, NGSadmix (included in ANGSD) was run using as input the genotype likelihood file for various k‐values.

### 2.6 Statistical analysis of phenotypes

All analyses were conducted in R (v4.3.0) within the RStudio (2022.02.3 + 492) environment. To summarize overall phenotypic differences across experimental settings, we performed principal component analysis (PCA) of the phenotypes using the prcomp (v3.6.2) package (https://www.rdocumentation.org/packages/stats/versions/3.6.2). All the boxplots were produced in R using the ggplot2 (v3.4.1) (https://github.com/tidyverse/ggplot2) and significant differences were calculated using the t‐test within the ggpubr (v0.6.0) package (Kassambara, 2023). Scatterplots were generated in ggplot2 for each comparison and Pearson correlation coefficients were calculated. Broad sense heritability was calculated for all settings using the inti (v0.6.3) package, which computes this statistic using Cullis and Piepho methods, which are well‐suited for studies with varying replication numbers because they incorporate mixed‐effects models that explicitly account for differences in replication across experimental units. These models allow for the accurate partitioning of variance components by estimating fixed and random effects separately (Lozano & Kistner, 2023).

### 2.7 GWAS analysis

The GWAS analysis was conducted by performing linear mixed model analysis using the likelihood ratio test in GEMMA (v0.98.5) (X. Zhou, 2017) and in GAPIT (a Genome Association and Predicted Integrated Tool) (v3.4) (J. Wang & Zhang, 2021). For GAPIT, the analysis was run using GLM (‘general’ linear model), MLM (Mixed linear model), MLMM (Multi‐locus mixed‐model), FarmCPU (Fixed and random model Circulating Probability Unification) and BLINK (Bayesian‐information and Linkage disequilibrium Iteratively Nested Keyway) methods. For both tools, to account for population stratification, we included 5 principal components from the PCAngsd output as covariates and the internally generated centered kinship matrix. For all quantitative traits, we conducted the analysis using both raw and quantile‐normalized phenotype distributions. To perform correction for multiple tests, we estimated the actual number of tested variants using the Genetic Type I error calculator and adjusted the suggestive and Bonferroni significance thresholds accordingly (M.‐X. Li et al., 2012). We also calculated the significance threshold based on False Discovery Rate (FDR)‐adjusted p‐values (van den Oord, 2008). We extracted regions upstream and downstream of high‐confidence SNPs in accordance with the calculated LD decay. We annotated those regions using a custom database generated with the QQ74 reference genome V1 (v1.4, id33827), using SnpEff 4.3 T in Galaxy (https://usegalaxy.org/) (Cingolani et al., 2012; The Galaxy Community, 2022). SNPs were categorized according to their position with respect to gene coding sequences. The putative functions of genes within the regions were predicted using online tools DAVID (https://david.ncifcrf.gov/tools.jsp) and Plaza (https://bioinformatics.psb.ugent.be/plaza) (Huang et al., 2009; Van Bel et al., 2022).

## 3 RESULTS

### 3.1 Root restriction allows prediction of field behavior

In the context of plant breeding and research, open field trials can yield direct insights into the agronomic performance of plants, but they are labor‐ and time‐intensive. On the other hand, controlled conditions can increase the repeatability of trials and control for specific factors, but the screening size is constrained by the experimental facility (e.g. illumination, space). In plants exhibiting shade‐avoidant behavior, such as quinoa, this limitation is exacerbated (Roig‐Villanova et al., 2019), which requires either high light intensity or a wide distance between plants. This makes large indoor screenings impractical. We hypothesized that growing quinoa using root restriction in a controlled environment would effectively reduce plant size and provide a reproducible framework for screening agronomic traits in a high‐throughput fashion. To validate the reliability of the system, we compared quinoa plants grown under controlled conditions in the presence or absence of root restriction with plants grown in an open‐field setting. The number of accessions used for each setting was variable and maximized based on space availability in order to have better insight into the distributions of phenotypes. We maintained a minimum overlap of 23 accessions between all settings, which was used for one‐to‐one comparative analyses (Figure S1b).

Root‐restricted plants were grown in a greenhouse in 100‐mL containers in two consecutive partially overlapping batches: restricted‐1 (winter 2021 with 69 accessions) and restricted‐2 (summer 2022 with 48 accessions). This allowed us to check for repeatability at different times of the year. As quinoa growth under controlled conditions is often carried out in 1 to 5‐L pots (Becker et al., 2017; Bois et al., 2006; Peterson et al., 2015; Tovar et al., 2020), 73 accessions were greenhouse‐grown in a single batch in 2‐L containers (standard size in our facility) during winter 2021. We defined these plants as grown under “standard conditions”. For the open field setting, we selected 36 accessions, which were then planted in April 2022 and harvested in October 2022 (depictions of the experimental settings are shown in Figure S3). To assess the plant performance during development and at maturity, we focused on 4 traits of agronomic interest: weeks to flowering, dry plant biomass, seed diameter, and total seed weight per plant.

**FIGURE 1:**
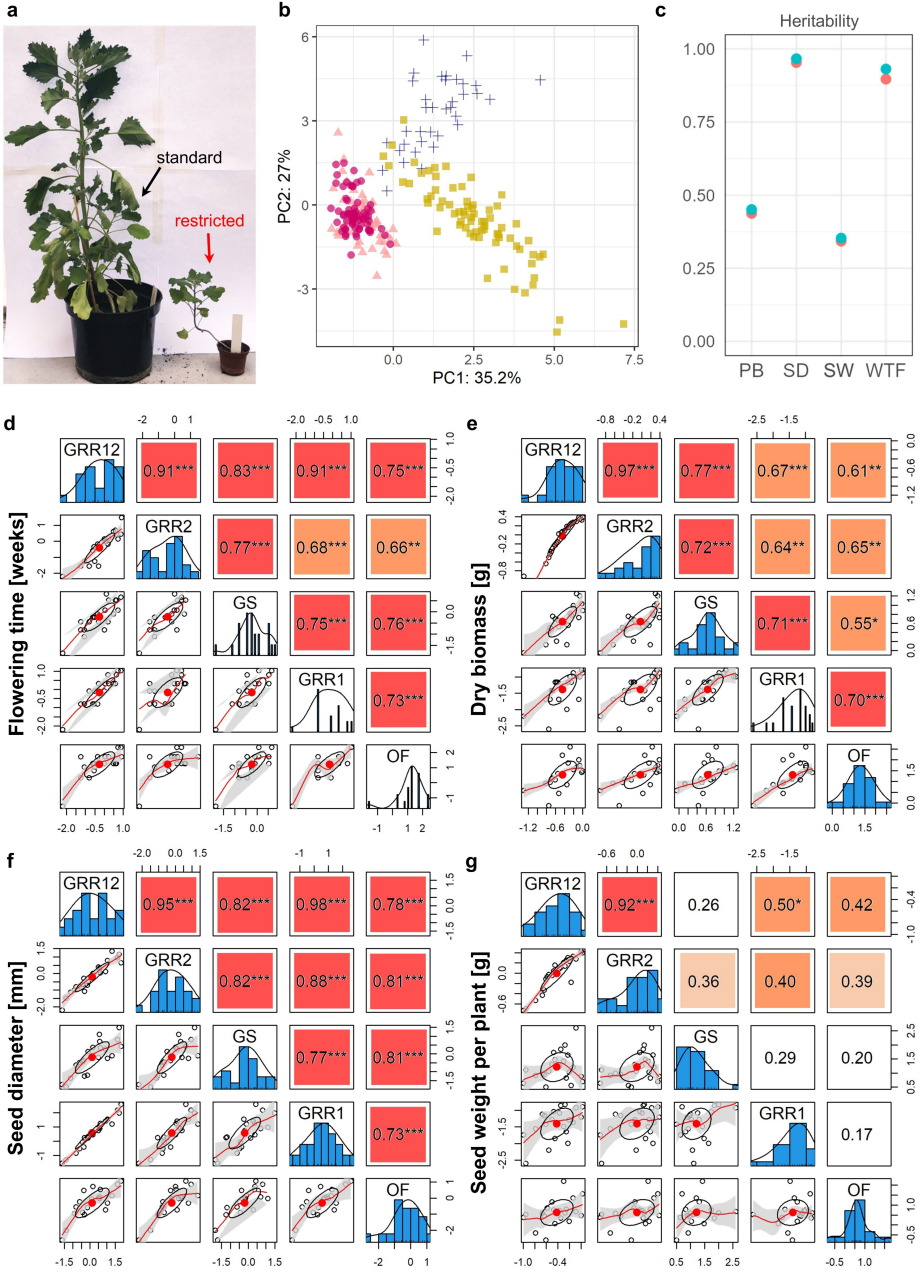
Impact of root restriction on plant phenotypes. (a) Danish line Titicaca grown in a standard 2‐L container and a root‐restriction container. (b) Principal component analysis for combined overview of phenotype scores across experimental settings. Red circles: restricted‐1, pink circles: restricted‐2, yellow squares: standard, blue crosses: field. (c) Broad sense heritability computed with Cullis (orange) Piepho (blue) methods. Abbreviations: Weeks to flowering (WTF), dry biomass post‐harvest (PB), total seed weight per plant (SW), seed diameter (SD). (d‐g) One‐to‐one comparison matrixes for the phenotypic scores between different settings. For each trait, scores histograms, scatter distributions, Lowess polynomial regression and correlation eclipses are plotted. Significant Pearson (* *p* < 0.05, ** *p* < 0.01, *** *p* < 0.001) coefficients are highlighted in colors (red >0.7, dark orange >0.4, light orange >0.2). Abbreviations: greenhouse restricted‐1 (GRR1), restricted‐2 (GRR2), restricted‐1/2 (GRR12) and standard (GS), open field (OF).

As expected, root restriction had a strong inhibitory effect on plant growth (Figure 1a). To get an overview of the overall behavior of plants in our different setups, we performed principal component analysis (PCA) with combined data from all accessions, traits and conditions. We observed that standard‐ and field‐grown plants formed two highly distinct clusters, while plants from restricted‐1 and restricted‐2 largely overlapped (Figure 1b). This indicated that while plants grown in the various settings are fundamentally different, root‐restricted plants behaved similarly between separate batches despite the different growing season, showing the usefulness of controlled conditions in reducing this source of variation.

The phenotype distributions for individual traits suggested that flowering time and seed size are less sensitive to growth conditions, while biomass and overall seed production are more affected by environmental factors (Figure S4a‐d). To quantify this effect, we computed broad sense heritability (the proportion of a trait that is genetically determined) using the subset of 23 accessions present in all settings (Lourenço et al., 2017; Schmidt et al., 2019) and Cullis and Piepho methods (Lozano & Kistner, 2023). Heritability calculated across all conditions showed that flowering time (H2 = 0.89–0.93) and seed diameter (H2 = 0.95–0.97) had a stronger genetic component than biomass (H2 = 0.44–0.45) and seed weight per plant (H2 = 0.34–0.35), confirming the results of the phenotypic analysis (Figures 1c and S5; Table S3). This was also true when considering setups in a pairwise fashion (Figure S4e‐h).

To assess the robustness of root restriction, we performed one‐to‐one comparisons of phenotypes from overlapping accessions grown in different experimental settings. Besides measurements from restricted‐1 and restricted‐2 plants taken individually, we calculated average scores between the two (restricted‐1/2) to compensate for the variation between different batches. Overall, root‐restricted plants performed similarly to standard plants in predicting field behavior (Figure 1d‐g). In particular, positive correlations (Pearson coefficient R) were observed for flowering time (R = 0.66–0.75, p‐value <0.001) and seed diameter (R = 0.73–0.81, p‐value <0.001). Furthermore, it was possible to see strong positive correlations for a trait with otherwise low heritability, like biomass (R = 0.61–0.70, p‐value <0.01–0.001). Notably, correlations were similarly high regardless of the experimental setting: restricted plants were just as effective in predicting behavior in other restricted batches as in standard or open‐field conditions. On the other hand, total seed weight showed higher variation between the settings (R = 0.17–0.42, non‐significant). In this respect, it is important to note that growth in larger containers did not provide any advantage since correlations remained low for plants grown under standard conditions. Interestingly, restricted‐1/2 data seemed to be slightly better at modelling field behavior than restricted‐1, restricted‐2, and standard plants. This hints at the clear benefit of repeated trials in screening studies in reducing environmental bias, which in controlled conditions can be facilitated by the smaller space constraints posed by plants under root restriction. Overall, we showed that despite introducing drastic morphological changes, root restriction is a robust strategy to increase screening throughput under controlled conditions, as it allows processing a larger number of plants while predicting field behavior with a reliability comparable to that of standard setups using larger containers.

### 3.2 Screening of a root‐restricted quinoa diversity panel reveals underlying population structure and selection patterns

After validating root restriction under controlled conditions as a predictor of field behavior, we aimed to further test the system for genomic studies by employing larger populations. For this, we obtained whole genome re‐sequencing data from a total of 124 accessions (see materials and methods). Of this panel, we phenotypically characterized 100 accessions in quadruplicates. To reduce the variation in traits with low heritability, we partially alleviated the root‐restriction conditions by using containers of 0.25 L in size.

In addition to the previous four agronomically relevant traits, we included two other parameters of interest: stem pigmentation (related to betalain content) and hundred‐kernel‐weight (all measurements are reported in Table S4 and Figure S6a‐f). Stem pigmentation in quinoa is caused by the accumulation of betalains, anthocyanin‐like compounds synthesized and allocated in various quinoa tissues and conferring them a coloration from yellow to purple (Gandía‐Herrero & García‐Carmona, 2013). We scored betalains produced in the stem of plants for several reasons. Firstly, we observed clear differences between individuals with different degrees of stem pigmentation, making this a relatively straightforward trait to score. Secondly, betalains have anti‐oxidant properties and are useful in several food and pharmaceutical sectors (Escribano et al., 2017). Betalain‐rich lines could potentially be reutilized as waste biomass after betalain extraction (Cai et al., 2005). Hundred‐kernel weight is a measure of seed size recorded as the weight of 100 seeds. This parameter is often used in agricultural research and crop management to assess seed quality and predict yield potential (Zhao & Su, 2019).

**FIGURE 2:**
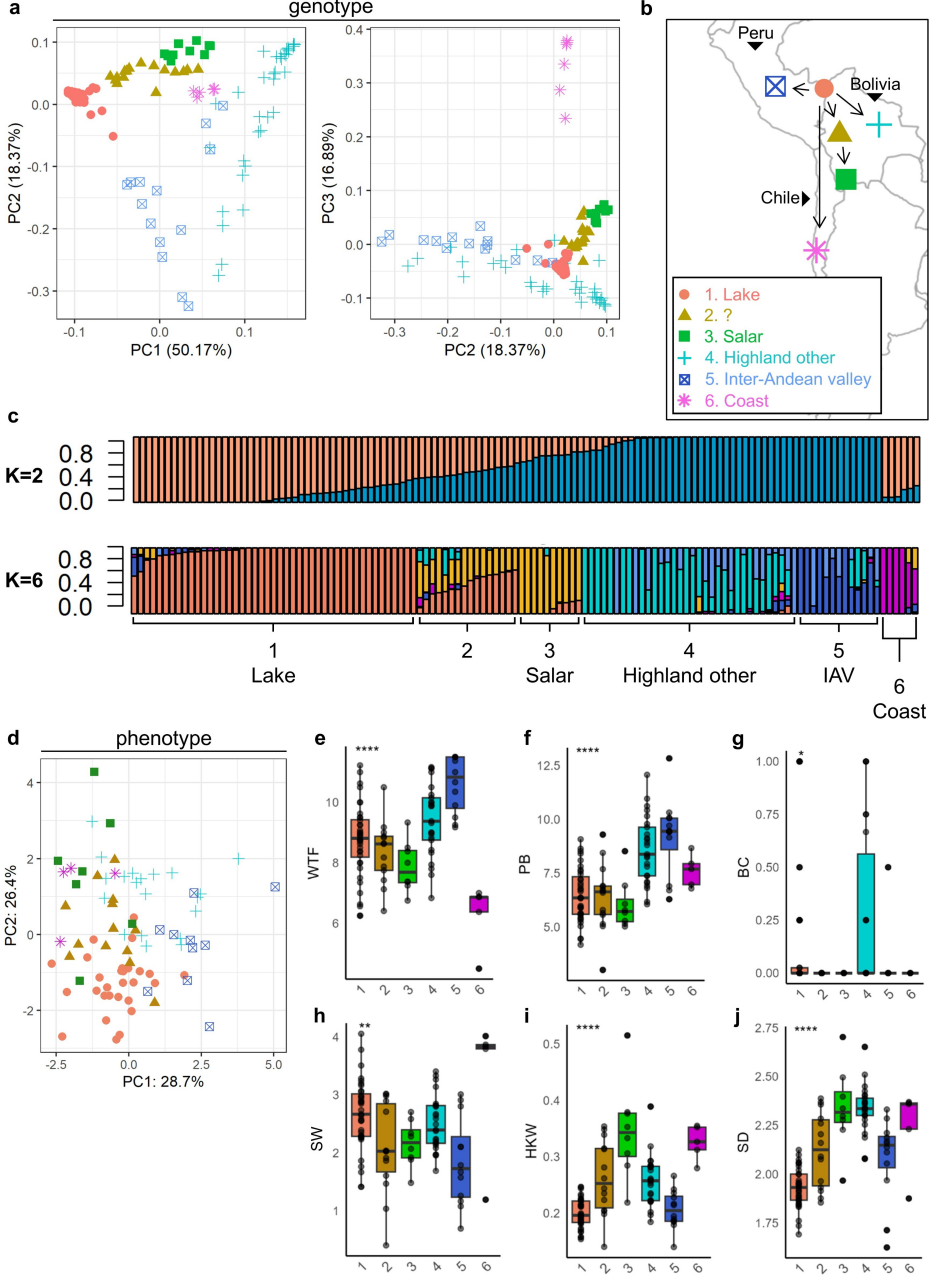
Characterization of a root‐restricted quinoa diversity panel. (a) Genotype likelihood‐based population structure, visualizing principal components PC1, PC2 (left) and PC2, PC3 (right). Colors and shapes refer to 6 sub‐population clusters identified by k‐means. (b) Suggested geographical origin of each germplasm subpopulation, based on k‐means clustering and existing IPK annotation. Symbols and colors match k‐means clusters. Arrows show previously described domestication trajectories. (c) Admixture analysis using k = 2 (highland, coastal) and k = 6 (k‐means clusters), performed across all 124 accessions used to construct the population structure. Numbers indicate putative k‐means sub‐populations, colors indicate NGSadmix groups. Abbreviations: Inter‐andean valley (IAV) (d) PCA of combined phenotypic scores. Colors and shapes match the k‐means clusters. (e‐j) For each trait: boxplots representing the distribution of phenotypes (y‐axis) across individuals within each k‐means cluster (x‐axis). Kruskal‐Wallis significance scores are reported (NS: non‐significant, * p < 0.05, ** p < 0.01, **** *p* < 0.0001). Abbreviations: Weeks‐to‐flowering (WTF), Plant dry biomass after harvest (PB) expressed in g, Stem betalain content (BC) scored between 1–5 for individual replicates and normalized on a scale from 0 to 1, Seed weight per plant expressed in grams (SW), Hundred‐kernel weight (HKW) expressed in g, Seed diameter (SD) expressed in mm.

To get an overview of the genetic diversity within our panel, we first looked at the genotype population structure using PCA. Besides a main subdivision between highland and coastal backgrounds, several putative ecotypes have been previously defined amongst highland accessions. The main ones are altiplano/highland (high‐altitude flats, also including accessions from the area near Lake Titicaca), salar (salt flats), yungas (tropical and humid regions), and inter‐andean valleys (variable climatic conditions at lower altitudes, higher humidity) (Murphy et al., 2018). To investigate the presence of sub‐populations within our panel, we performed unsupervised clustering on the first 5 principal components using k‐means, which yielded an optimal number of 6 groups (Figure S7a‐c). The classification from k‐means identified 5 putative sub‐populations among highland accessions and 1 likely belonging to the coastal group (Figure 2a). Notably, the division between highland and coastal lines was only visible in the distribution by plotting the 3rd principal component, which explained ~17% of the variation. This was due to the low number of accessions in our panel that appeared to belong to this group (5 accessions).

To get insights into the geographical origin of accessions, we first retrieved the metadata on germplasm geographical origin registered at the IPK Gatersleben Gene Bank (Figure S7d). This information is incomplete and only refers to the place where seeds were acquired/purchased and not necessarily their geographical origin. We reconstructed the putative geographical origins by merging the result of the clustering with the available annotation (Figure 2a,b). A total of 45 accessions seemed to belong to the area close to La Paz, near Lake Titicaca, which is the domestication epicenter for all highland accessions (Bazile et al., 2013). Most of these lake lines clustered closely together. We also identified a cluster of 10 accessions possibly belonging to the salar ecotype, as the only two germplasm samples from Bolivia salt flats belonged to this group (Figure S7d). These were found relatively near the lake group. Between these two, there was a third cluster of 16 accessions, which was not clearly separated from the others. The remaining accessions were subdivided into unspecified highland (33 accessions) and inter‐andean valleys (14 accessions) and clustered further away from the domestication origin.

While there were some clear separations between groups, there also seemed to be a certain degree of admixture between them. Indeed, the k‐means average silhouette was 0.52 (Supplementary figure S7c), indicating a probable overlap between clusters. This intersection can be quantified at the genetic level using admixture analysis. We used NGSadmix and input number of subpopulations of k = 2 and k = 6, reflecting the subdivision between highland and coastal lines, and between the 6 groups identified by k‐means, respectively (Figure 2c). We observed that with k = 2, no clear subdivision was found between highland and coastal lines. This indicated that underlying groups were indeed present within highland accessions and that a certain degree of admixture could exist between our coastal and highland groups. On the other hand, when using k = 6, we observed a strong match between the k‐means predicted clusters and NGSadmix sub‐populations. As expected, lines from cluster 1 (lake) and 6 (coastal) mainly comprised non‐admixed individuals. Among the other groups, group 3 (salar) was also separated from the others to some degree. Group 2, on the other hand, was entirely comprised of accessions that presented a genetic contribution from both lake and salar, indicating that the k‐means subdivision was indeed correct. As observed from the clustering output, groups 4 (highland others) and 5 (inter‐andean valley) represented separate subpopulations, which had little shared genetic background with all the other groups.

To investigate the relationships between subpopulations at the phenotype level, we performed a PCA using the scores from the 6 selected phenotypic traits: weeks‐to‐flowering, dry biomass, betalain content, total seed weight per plant, hundred‐kernel weight and seed diameter (Figure 2d). The first two PCs cumulatively explained 52.5% of the variation. In this case, no clear clusters emerged. Interestingly, lake accessions, which clustered very closely together in the genotype‐based PCA, showed high phenotypic heterogeneity and increased overlap with other accessions. This shows that the genetic diversity in quinoa is high, even for closely related accessions.

To assess to what extent phenotypic data from root‐restricted plants can describe underlying genetic relationships, we investigated the phenotype distributions of single subpopulations from our population structure analysis in relation to others. Firstly, we could observe patterns of selection for flowering time (Figure 2e). In particular, lake accessions had a wide distribution of flowering time, which is expected for the origin of domestication and reflects the diversity of environments and latitudes found in the region around Lake Titicaca. On the other hand, salar and coastal lines flowered early, in line with their adaptation to higher latitudes. Conversely, inter‐andean valley lines flowered the latest, reflecting their domestication history closer to the equator. Similar patterns were observed for dry biomass (Figure 2f), which was weakly positively correlated with flowering time (Figure S8). We did not detect strong patterns for stem pigmentation (Figure 2g), albeit plants from group 4 seemed to produce, on average, more betalains in the stem. Coastal lines showed on average a higher total seed weight per plant than other groups (Figure 2h). Indeed, this group contained the Danish lines Vikinga and Titicaca, as well as other high‐latitude‐adapted accessions, which were expected to perform better under our experimental conditions. Interestingly, there were differences among subpopulations for traits related to seed size (hundred‐kernel weight and seed diameter) (Figure 2i,j). In particular, lines belonging to the subpopulations more distant from the domestication origin had significantly larger and heavier seeds. We interpret this as evidence of selection for increasingly larger seeds over the course of quinoa dispersion and domestication.

### 3.3 GWAS analysis identified loci significantly associated with key agronomic traits

Lastly, we combined the information from whole‐genome variant calling and phenotypes to investigate the genetic basis of the scored traits. For this, we performed variant calling to carry out a GWAS analysis. Our variants datasets contained 3,637,746 high‐confidence Single Nucleotide Polymorphisms (SNPs) (~2.6 SNPs/kb on average). SNP density was variable within chromosome scaffolds but similar between different chromosomes (Figure S9a). The global linkage disequilibrium (LD) decay measured at *r* ^*2*^ = 0.2 was determined to be around 40 kb (Figure S9b), which was consistent with previous findings for a diversity panel of quinoa (Patiranage et al., 2022). The Genetic type I error calculator outputted 933,620 (26%) true independent tests out of 3,637,746 markers. The resulting Suggestive p‐value was 1.07E‐6 [−log10(p) = 5.97], while the significant adjusted Bonferroni p‐value was 5.36E‐8 [−log10(p) = 7.27]. For quantitative traits (all traits except stem pigmentation), we performed the analysis using both raw phenotype data and quantile‐normalized data as input (Table S4; Figure S10). We observed that 2 out of 6 traits presented marker‐trait associations (MTAs) above Bonferroni threshold. These were stem pigmentation (47 SNPs in 15 loci) and seed diameter (raw: 5 SNPs in 3 loci, quantile‐normalized: 16 SNPs in 3 loci).

**FIGURE 3:**
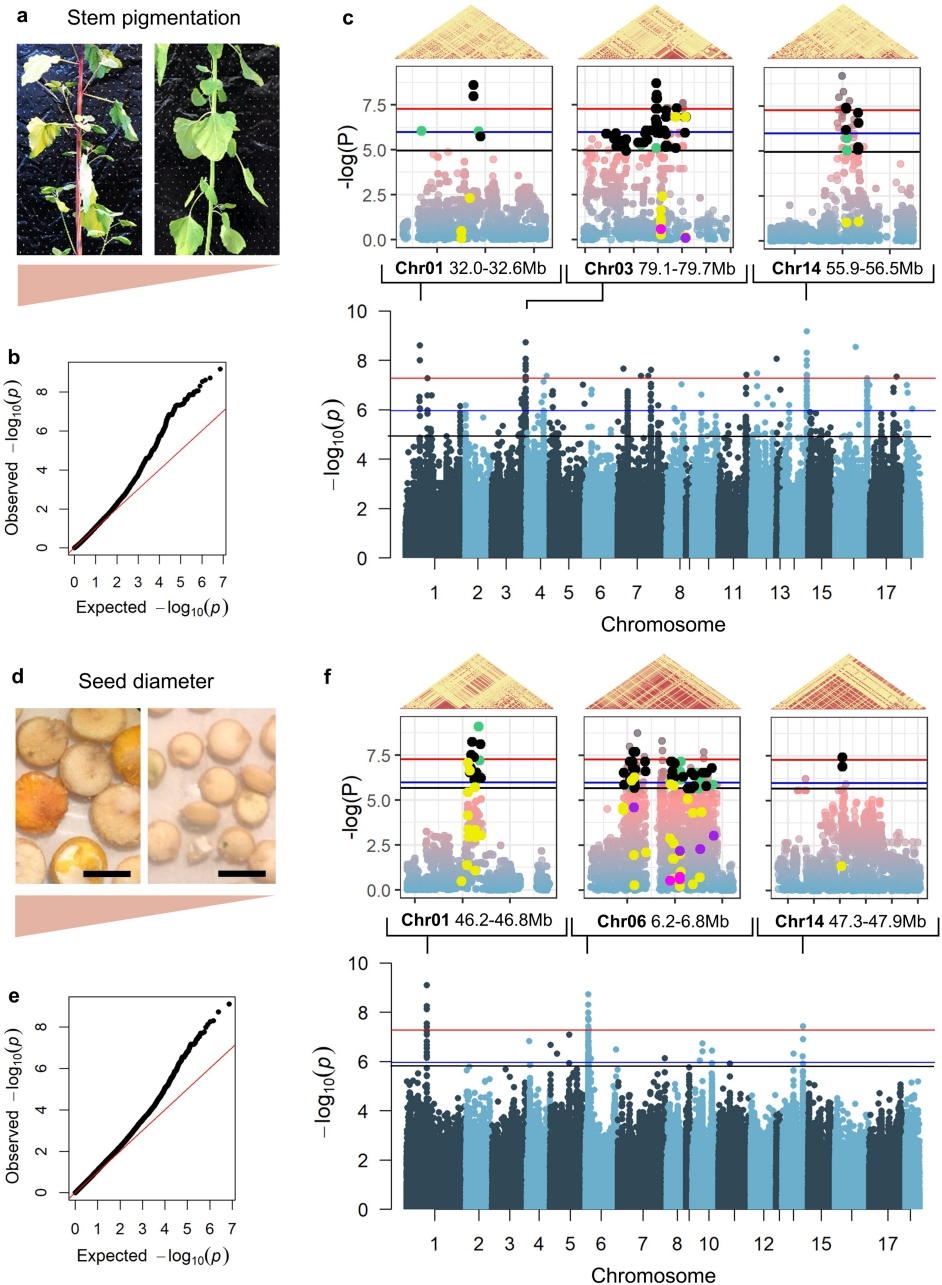
GWAS analysis by linear mixed model analysis using the likelihood ratio test in GEMMA. (a) Betalain content in stem (BC) was scored by visually assessing stem pigmentation. (b) Quantile‐quantile plot for stem pigmentation association analysis, comparing the distribution of observed p‐values against an expected normal distribution. (c) Manhattan plot for stem pigmentation association analysis depicting association p‐values as ‐log10(P) for all individual SNPs across the 18 chromosomes. Red and blue lines represent the Bonferroni and suggestive significance thresholds, respectively, adjusted for the actual number of independent tests. Black line represents the significance threshold for False Discovery Rate (FDR)‐adjusted p‐values. Local Manhattan plots of loci of interest are also shown. SNPs found within gene coding sequences are highlighted as follows: nonsense (magenta), missense (yellow), splicing (purple). Among the SNPs lying upstream/downstream (black) or in introns (green), only the ones above FDR significance threshold are shown. Linkage disequilibrium (LD) heatmaps for each region are included above the graphs, with red representing higher LD. (d) Quinoa accessions showing contrasting seed diameter (SD). (e) Quantile‐quantile plot for seed diameter association analysis. (f) Manhattan plot for seed diameter association analysis.

Concerning stem pigmentation, some plants exhibited a strong coloration of the stem, while, for others, the stem was almost entirely green (Figure 3a). We identified 65 SNPs located in 15 loci above the Bonferroni significance threshold (Table S5). However, we observed an inflation of lower p‐values compared to the expected distribution (Figure 3b), so we decided to focus the analysis on 3 main peaks above the Bonferroni threshold, located on chromosome 01, chromosome 03 and chromosome 14 (Figure 3c; Table S6). Notably, the locus in chromosome 01 was also found by BLINK and MLMM, while the locus in chromosome 03 was identified with BLINK, MLMM and FarmCPU (Figure S11a). Within these regions, we found a total of 29 annotated genes within +/− 40 kb of SNPs lying above the Bonferroni threshold, including several close homologs of genes coding for proteins belonging to families that can have roles in the betalain or flavonoid biosynthetic pathway, such as an UDP‐galactose/UDP‐glucose transporter, two MYBL2‐like transcription factors, and the sucrose transport protein SUC3 (Dubos et al., 2008; Hans et al., 2004; Schmitz et al., 2002; Stracke et al., 2014; Winkler et al., 2024; Xie et al., 2023; X. Zhang et al., 2022; J. Zhou et al., 2020). Gene variants included 1 nonsense, 20 missense and 2 splice‐site mutations, along with 102 intron variants (Figure 3c; Tables S7 and S9). Among these variants, we saw mostly dominant phenotype distribution patterns (Figure S12).

Additionally, on chromosome 03, we identified a close homologue of *Beta vulgaris BvDODA1* (*AUR62012187*), which is a key gene in the betalain biosynthesis pathway (Bean et al., 2018). A *BvDODA1* homologue associated with betalain score was also identified in the same region by Patiranage et al. (2022). A 5′ variant was significantly associated with the phenotype (Figure S12), and the presence of this gene further hinted at a betalain gene hub in this locus.

Concerning seed diameter, we observed a strong visible variation in this trait across our biodiversity panel (Figure 3d). To assess these differences in a quantitative fashion, seed diameter was measured by imaging and analyzing hundreds of seeds per single replicate plant. Similarly to stem pigmentation, the quantile‐quantile plot indicated a good match for low‐scoring SNPs but presented a progressive inflation of p‐values above –log10(p‐value) ~3 (Figure 3e). For this reason, we only focused on the 3 main peaks above the Bonferroni threshold found on chromosomes 01, 06, and 14 (Figure 3f; Table S6). Interestingly, the locus on chromosome 01 was also identified by FarmCPU and BLINK methods (Figure S11b). Within these regions, we found 29 annotated genes within +/− 40 kb of SNPs lying above the Bonferroni threshold, harboring a total of 3 nonsense, 39 missense and 4 splice site mutations, along with 207 intron variants (Figure 3f; Tables S8 and S9). Unlike stem pigmentation, we saw both strong additive and dominant phenotype distribution patterns among these variants (Figure S13). Notably, some of the annotated genes are homologues of genes linked to seed size and development in other plants, such as *Arabidopsis PEX1* (*AUR62024782*) (Luan et al., 2022), rice/wheat *MGD A* (*AUR62024785*) (Basnet et al., 2019; Du et al., 2020), and *Jatropha IAA9* (*AUR62032874*) (Sun et al., 2017; Ye et al., 2014). We also identified several genes with unknown functions that could possibly be associated with the trait.

## 4 DISCUSSION

The screening of plant populations coming from biparental/multiparental crosses, random mutagenesis, or diversity panels represents a technical and logistical challenge in plant science and plant breeding. While the characterization of plants in different environments and times is extremely important, the resources for large screening trials are often limited, especially when it comes to underutilized crops such as quinoa. This study analyses the potential of root‐restricted plants as a tool to increase phenotyping throughput under controlled conditions. In a breeding context, assessing plant behavior under root restriction in greenhouses could help narrow down the pool of candidate varieties used for detailed multi‐environment field trials. This approach could help select promising subsets of plants from larger groups and optimize resources. Besides plant breeding, such a method could be suitable for increasing sample size in research setups, which are more often associated with controlled conditions.

Root restriction inhibits plant growth and reduces space constrains but it also affects plant architecture, chlorophyll content, and photosynthetic activity, as well as uptake of water and nutrients (Ismail & Davies, 1998; Kemble et al., 1994; NeSmith & Duval, 1998). For this reason, while this method is useful for horticulture applications, it is unclear how robust it is when it comes to producing observations that are transferable to field contexts. In this work, we phenotyped a panel of quinoa accessions for four key agronomical traits and compared data from controlled settings (in presence or absence of root restriction) with field‐grown plants.

As expected, inhibition of plant growth under root restriction was reflected in the reduced dry biomass measured after harvest. Plants grown under standard conditions also accumulated lower biomass than their field counterparts, which explains the low heritability of this trait. Strong variations in the heritability estimates for biomass‐related parameters (height, dry weight) are found across previous studies, with some reporting values of ~30–40% (De Santis et al., 2016; Maldonado‐Taipe et al., 2022), and others finding higher values (> 70%) (Al‐Naggar et al., 2017; Hafeez et al., 2022; Patiranage et al., 2022). It is important to note that, in contrast to the bulkier and bushier plant architecture observed in the field for several accessions, both restricted and standard‐grown plants presented an elongated morphology with an apical panicle, with this phenotype being more pronounced in restricted plants. This could explain the variation observed for total seed yield between plants grown in field, standard, or root‐restricted conditions. In contrast, the two plant batches grown in the same setting (restricted‐1 and restricted‐2) showed comparable total seed weight per plant. This is in line with the high heritability found for this trait across different field‐grown batches in previous studies (Al‐Naggar et al., 2017; Maldonado‐Taipe et al., 2022). Flowering time and seed size were not strongly affected by the container size. Indeed, it is known that both of these traits are mostly influenced by photoperiod, while other factors such as biomass, humidity, temperature or nutrient availability have an overall more modest impact (Al‐Naggar et al., 2017; Bertero et al., 1999; Christiansen et al., 2010; De Santis et al., 2016; Maldonado‐Taipe et al., 2022; Patiranage et al., 2022). While our data indicates that root restriction can be used to predict field behavior for specific traits, there are clear limitations. For instance, in the screening of architecture‐related traits or traits with a complex below‐ground component (such as total seed weight in our setup). However, such limitations are also present for standard‐grown plants under controlled conditions, which have the disadvantage of requiring a larger space.

After showing the possibility of obtaining reliable phenotyping data from root‐restricted plants for certain relevant agronomical traits, we set out to demonstrate how this could be used in a real screening scenario. For this, we assessed the genetic and phenotypic variation within a quinoa diversity panel. Concerning the genotype data, our population structure results are in general agreement with the existing literature (Patiranage et al., 2022). Interestingly, we identified a cluster of accessions with a low degree of admixture that could belong to the salar ecotype. This putative classification is strengthened by previous evidence that found how salar accessions can be genetically separated within highland accessions (Christensen et al., 2007). The group of lake accessions was composed primarily of closely related samples. However, they showed remarkable phenotypic diversity. This underscores the important difference between genetic relatedness and genetic heterogeneity, showing how highland‐lake accessions present a rich hub of phenotypic diversity that can be easily accessed by screening under severe root restriction. We also uncovered patterns of selection for several traits, particularly for flowering time and seed size/weight. Taken together, these results show how root restriction makes it possible to screen large plant panels and can yield reliable and deep insights into quinoa heterogeneity, biology, and domestication history.

To further illustrate the possibilities of the root restriction system, we combined whole‐genome sequencing data with phenotypic scores to identify candidate genetic loci for traits of interest. While we did see SNPs above the suggestive threshold for all traits tested (Figure S10), only betalain content and seed diameter presented highly significant loci. We attribute the lack of strong signal across other traits to the reduced size of our panel, which affected the power of the analysis, as well as to the quantitative nature of these traits. While seed diameter is also a quantitative trait, the segregation of seed size in our panel was very marked, increasing the power of our statistical tests and allowing us to pinpoint significant MTAs. A way to strengthen the found associations for the other traits would be to increase the number of accessions to screen. Another possible approach could be to repeat the analysis with another reference genome version. At the time when we carried out our analysis, only the first version of the quinoa reference genome was available, while a new updated version was later published (Rey et al., 2023, 2024). The updated reference genome could yield more accurate results, as more variants would be mapped correctly into the corresponding chromosome blocks, and LD ambiguity arising from structural variation could be minimized.

Concerning stem pigmentation, a previous study identified a locus on chromosome 1B (Chr03 in our genome version) associated with plant pigmentation and containing a homologue of betalain gene *BvDODA1* (Patiranage et al., 2022). We also identified several MTAs in the same region, as well as significantly associated variants in the 5′ regulatory region of a *BvDODA1* homologue. This showed the possibility of reproducing results from existing studies by scoring plants in restricted conditions. Notably, none of the 3 major loci found in our studies completely explained the phenotype distribution, suggesting other underlying contributions. This is in accordance with current knowledge, as several genes contribute to betalain production (Gandía‐Herrero & García‐Carmona, 2013). Also, betalains biosynthesis is strongly linked to abiotic stress responses, which implies that replicate plants with the same genetic background can exhibit different coloration patterns depending on the specific growing conditions (Ain et al., 2023). Indeed, despite our study being performed under controlled conditions, we observed high heterogeneity in stem pigmentation scores across positive accessions. Future work should focus on the genes responsible for betalain synthesis across different tissues as well as identifying possible master regulators for this biosynthetic pathway in quinoa.

Finally, we identified candidate loci for seed diameter. Grain yield and grain size hold significant importance and represent crucial traits to increase quinoa productivity and marketability (Bertero et al., 2004). In our study, we were able to identify several variants strongly associated with segregation in seed diameter. While the population structure analysis showed a stratification for this trait, we accounted for this in our GWAS analysis to avoid biases. Interestingly, no significant SNPs were found for hundred‐kernel weight, even though the trait was correlated with seed diameter (Figure S8). This could be due to the fact that seed diameter was directly derived from image data and averaged across tens to hundreds of seeds, while hundred‐kernel weight was calculated based on seed particles counted in the same image and the corresponding recorded weight on a laboratory scale, cumulatively combining two sources of uncertainty. Increasing the sample size or improving the imaging setup could help reduce this variation. Our findings are relevant and promising for future work on molecular targets to increase seed size in quinoa in an agronomic context. In accordance with this, we did not observe a negative interaction between total seed weight and both seed diameter and hundred‐kernel weight (Figure S8). This points to the previously suggested possibility of improving quinoa seed‐related traits without negatively affecting yield (Bertero et al., 2004).

## 5 CONCLUSIONS

In plant science, field trials require time, an appropriate climate, large cultivation spaces, and extensive manpower in order to succeed. The advantages of using controlled conditions stretch from repeatability to the possibility of controlling experimental parameters. Root restriction allows the design of experimental strategies with a larger number of plants. Root‐restricted plants can be easily kept and propagated in climate chambers, facilitating the overall workflow with plants of a large size, such as quinoa. For suitable traits, pre‐screening of large plant variety libraries could be conducted under controlled conditions before further characterizing and assessing a subgroup of plants in the field. Using the method, we conducted a screening of a quinoa diversity panel. We were able to validate previously identified loci for betalains biosynthesis while also identifying MTAs for betalain content and even for a quantitative trait such a seed diameter. In conclusion, the root‐restriction setup presented here could provide a useful framework for phenotyping quinoa under controlled conditions and substantially accelerate the improvement of this remarkable crop.

## AUTHOR CONTRIBUTIONS

DV and RLLM developed the concept and designed the article. DV designed experiments, collected and analyzed data, prepared and edited figures and tables and wrote the first version of the manuscript, which was improved with the help of RLLM. NFS and MC collected phenotyping data for restricted‐1 and restriced2 plants. MDLT provided expert guidance on plant growing conditions and propagated seeds that were used in this work. RRF and SFA provided expert guidance and support for genomic data analysis and critically reviewed the manuscript. RLLM supervised the project. All the authors have read and agreed to the published version of the manuscript.

## FUNDING INFORMATION

This project has received funding from the Novo Nordisk Foundation (Project number NNF19OC0056580/NovoCrops) and the European Union's Horizon 2020 research and innovation program under the Marie Skłodowska‐Curie grant agreement No 801199.

## Supporting information


**Figure S1:** Field sowing scheme and upset plot of experimental settings.
**Figure S2:** Imaging setup for calculation of seed diameter and counting.
**Figure S3:** Experimental setup for testing reliability of root restriction.
**Figure S4:** Phenotypic scores and Heritability comparisons between experimental conditions.
**Figure S5**: Residual plots for fixed and random effect models.
**Figure S6:** Distributions of traits scored in a quinoa diversity panel.
**Figure S7:** k‐means clustering of population structure.
**Figure S8:** Correlations between scored traits, including population structure.
**Figure S9:** SNP density and linkage disequilibrium decay.
**Figure S10:** GWAS analysis, GEMMA output for of all traits.
**Figure S11:** GWAS analysis, GAPIT methods output.
**Figure S12:** Stem pigmentation, phenotype distributions for SNPs of interest.
**Figure S13:** Seed diameter, phenotype distributions for SNPs of interest.
**Table ST1:** All accessions and measurements for root restriction testing.
**Table ST2:** All accessions identifiers used to build population structure.
**Table ST3:** Broad sense Heritability estimates between all experimental conditions.
**Table ST4:** Phenotyping raw and normalized scores, averaged between replicates.
**Table ST5:** GWAS analysis, SNPs and loci number laying above the 3 significance thresholds.
**Table ST6:** SNPs above Bonferroni threshold (negLogP = 7.27).
**Table ST7:** Stem pigmentation: Genes within +/−40kb of most significant SNPs (negLogP > 7.27 Bonferroni).
**Table ST8:** Seed diameter: Genes within +/−40kb of most significant SNPs (negLogP > 7.27 Bonferroni).
**Table ST9:** Functional annotation of variants found within gene regions.

Data S1

## Data Availability

All data and code generated during this study and not included in supplementary material can be found in the following links: Raw Illumina reads: https://sid.erda.dk/share_redirect/bh8CDs585r https://sid.erda.dk/share_redirect/e68mXhVpv8 https://sid.erda.dk/share_redirect/aN1stPXzLR https://sid.erda.dk/share_redirect/b0jSukNWKO https://sid.erda.dk/share_redirect/fQGtvs10Xs Variant set files: https://sid.erda.dk/sharelink/kSuz8rD8NL Population structure: https://sid.erda.dk/sharelink/OW7MrARkan Phenotype scores data: https://sid.erda.dk/share_redirect/BfCq3RwJZS
