# Supplementary material for: Root restriction accelerates genomic target identification in quinoa under controlled conditions
Source: Physiol Plant. 2025 Apr 15;177(2):e70223. doi: 10.1111/ppl.70223 (PMC11998636; doi:10.1111/ppl.70223)
Supplement: Supplementary file 1 — Figure S1: Field sowing scheme and upset plot of experimental settings. Figure S2: Imaging setup for calculation of seed diameter and counting. Figure S3: Experimental setup for testing reliability of root restriction. Figure S4: Phenotypic scores and Heritability comparisons between experimental conditions. Figure S5: Residual plots for fixed and random effect models. Figure S6: Distributions of traits scored in a quinoa diversity panel. Figure S7: k‐means clustering of population structure. Figure S8: Correlations between scored traits, including population structure. Figure S9: SNP density and linkage disequilibrium decay. Figure S10: GWAS analysis, GEMMA output for of all traits. Figure S11: GWAS analysis, GAPIT methods output. Figure S12: Stem pigmentation, phenotype distributions for SNPs of interest. Figure S13: Seed diameter, phenotype distributions for SNPs of interest. Table ST1: All accessions and measurements for root restriction testing. Table ST2: All accessions identifiers used to build population structure. Table ST3: Broad sense Heritability estimates between all experimental conditions. Table ST4: Phenotyping raw and normalized scores, averaged between replicates. Table ST5: GWAS analysis, SNPs and loci number laying above the 3 significance thresholds. Table ST6: SNPs above Bonferroni threshold (negLogP = 7.27). Table ST7: Stem pigmentation: Genes within +/−40kb of most significant SNPs (negLogP > 7.27 Bonferroni). Table ST8: Seed diameter: Genes within +/−40kb of most significant SNPs (negLogP > 7.27 Bonferroni). Table ST9: Functional annotation of variants found within gene regions. [file PPL-177-e70223-s002.pdf]

Supplementary material

Root restriction accelerates genomic target identification in quinoa under controlled conditions

Davide Visintainer[1], Nanna Fjord Sørensen[1], Mengming Chen[1], Mai Duy Luu Trinh[1], Rute R. da Fonseca[2] , Sara Fondevilla Aparicio[3], Rosa L. López-Marqués[1,*]

Supplementary figures

Figure S1: Field sowing scheme and upset plot of experimental settings.
Figure S2: Imaging setup for calculation of seed diameter and counting.
Figure S3: Experimental setup for testing reliability of root restriction.
Figure S4: Phenotypic scores and Heritability comparisons between experimental conditions.
Figure S5: Residual plots for fixed and random effect models.
Figure S6: Distributions of traits scored in a quinoa diversity panel.
Figure S7: k-means clustering of population structure.
Figure S8: Correlations between scored traits, including population structure.
Figure S9: SNP density and linkage disequilibrium decay.
Figure S10: GWAS analysis, GEMMA output for of all traits.
Figure S11: GWAS analysis, GAPIT methods output.
Figure S12: Stem pigmentation, phenotype distributions for SNPs of interest
Figure S13: Seed diameter, phenotype distributions for SNPs of interest.

Supplementary tables

Table ST1: All accessions and measurements for root restriction testing
Table ST2: All accessions identifiers used to build population structure
Table ST3: Broad sense Heritability estimates between all experimental conditions
Table ST4: Phenotyping raw and normalized scores, averaged between replicates
Table ST5: GWAS analysis, SNPs and loci number laying above the 3 significance thresholds
Table ST6: SNPs above Bonferroni threshold (negLogP = 7.27)
Table ST7: Stem pigmentation: Genes within +/-40kb of most significant SNPs (negLogP > 7.27 Bonferroni).
Table ST8: Seed diameter: Genes within +/-40kb of most significant SNPs (negLogP > 7.27 Bonferroni).
Table ST9: Functional annotation of variants found within gene regions.

a

	3 m	0.5 m	3 m	0.5 m	3 m	0.5 m	3 m	0.5 m	3 m	0.5 m	3 m	0.5 m	3 m
	Titicaca												
0.5 m													
	222		253		262		628		593		183		369
0.5 m													
	636		638		208		218		Titicaca		307		143
0.5 m													
	161		191		635		Titicaca		131		154		469
0.5 m													
	644		185		Titicaca		177		225		138		Titicaca
0.5 m													
	345		Titicaca		133		226		219		624		176
0.5 m													
	Titicaca		136		178		205		115		Titicaca		213
0.5 m													
	211		214		360		243		195		472		Puno
0.5 m													
	Titicaca												

b

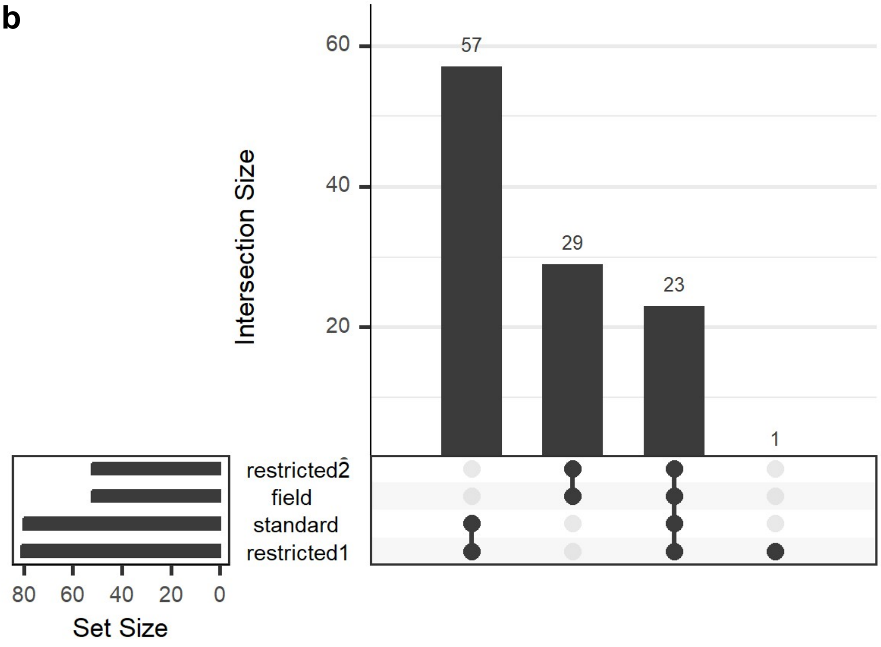

Figure S1: Experimental scheme supporting information a. Field sowing scheme. Titicaca rows are highlighted in blue. Rows length and distances are reported. **b.** Upset plot showing overlap between accessions sown in different settings.

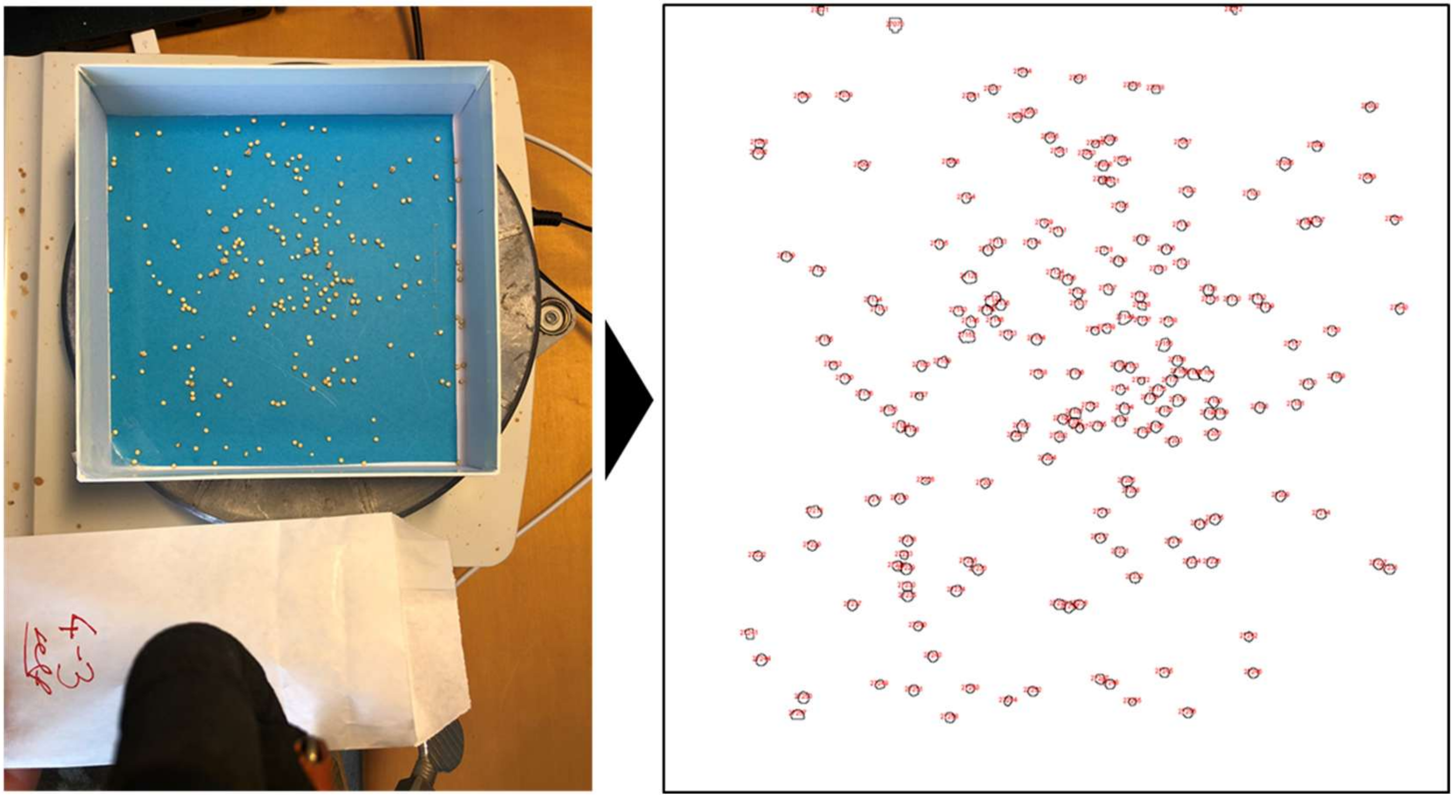

Figure S2: Imaging setup for calculation of seed diameter and counting. For each individual sample, seeds were imaged on top of a scale and the diameters and number of seeds were calculated in imageJ.

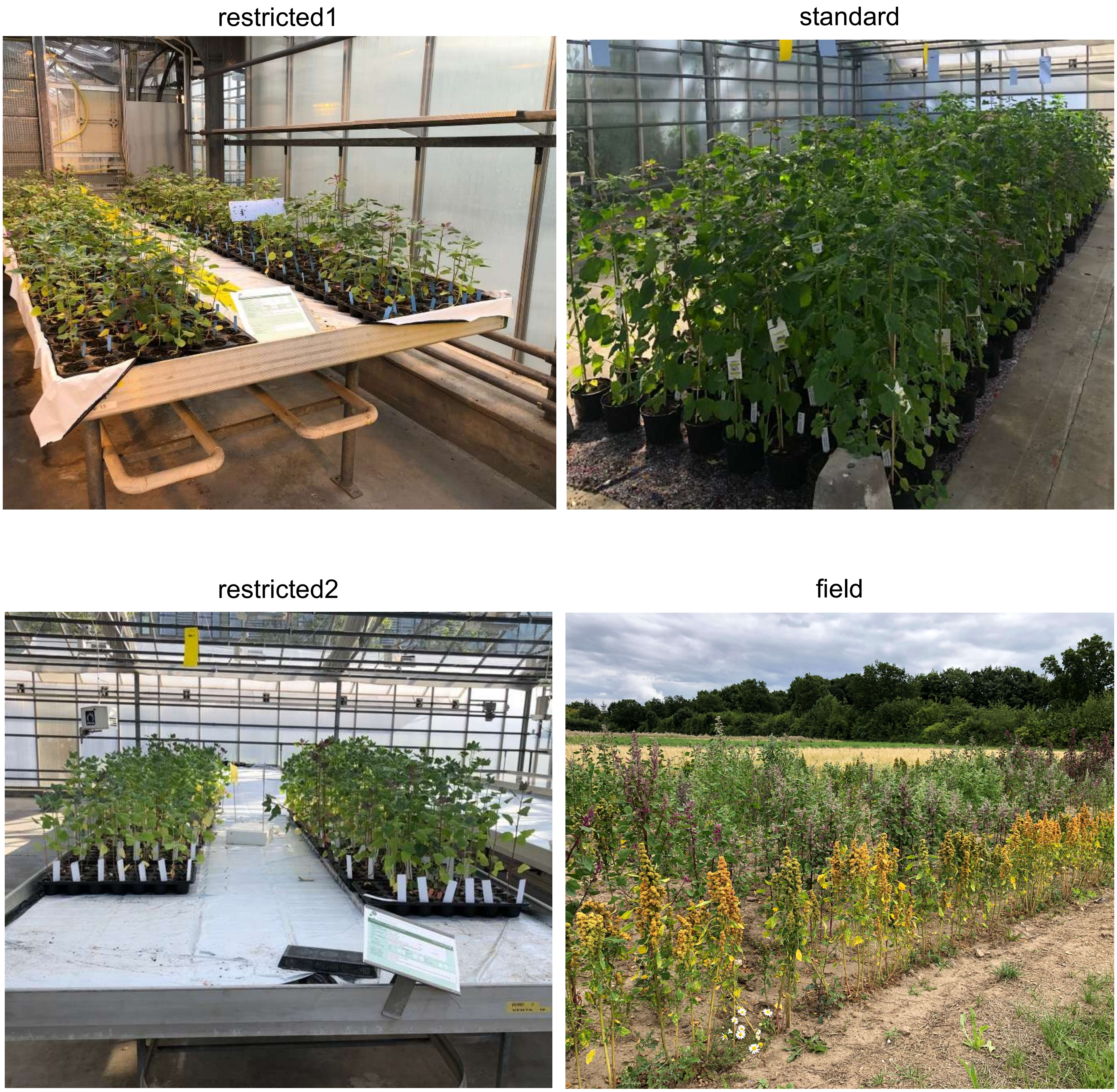

Figure S3: Experimental setup for testing reliability of root restriction. Restricted1 and restricted2 plants were grown in 2 consecutive batches. Standard plants were grown in a dedicated greenhouse room. Field plants were grown in the field during growing season.

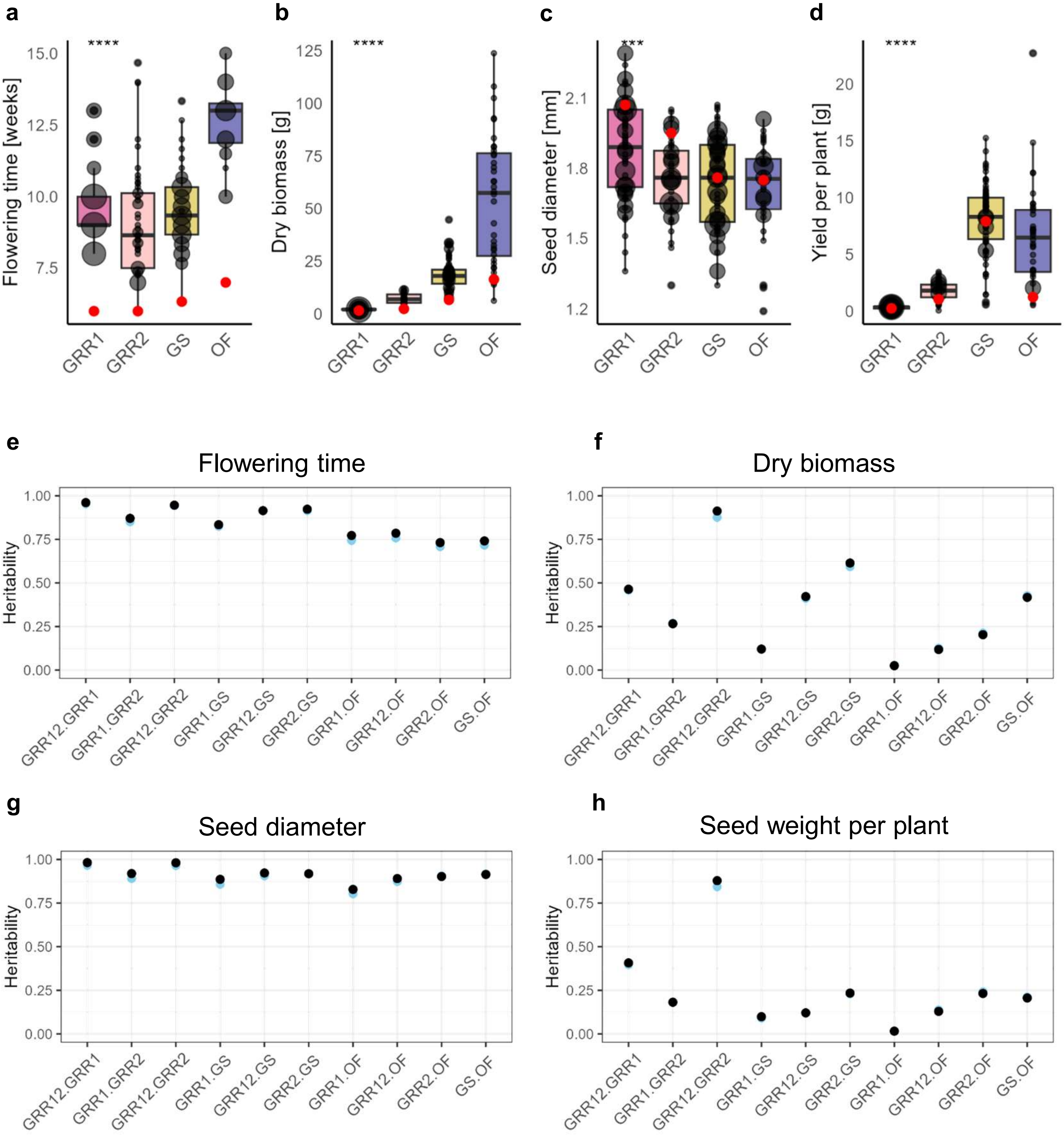

Figure S4: Phenotypic scores and Heritability comparisons between experimental conditions. a-d. Scores distributions for flowering time, dry biomass, seed diameter and average total seed weight per plant. Abbreviations: greenhouse: restricted-1 (GRR1), restricted-2 (GRR2), restricted-1/2 (GRR12), standard (GS). Abbreviation open field: field (OF). Red data points highlight the score for Danish-adapted line Titicaca. Kruskal-Wallis significance scores are reported (NS: non-significant, * p<0.05, ** p<0.01, **** p<0.0001). **e-h.** Heritability calculated between each two given settings. Heritability method: Cullis (light blue), Piepho (black).

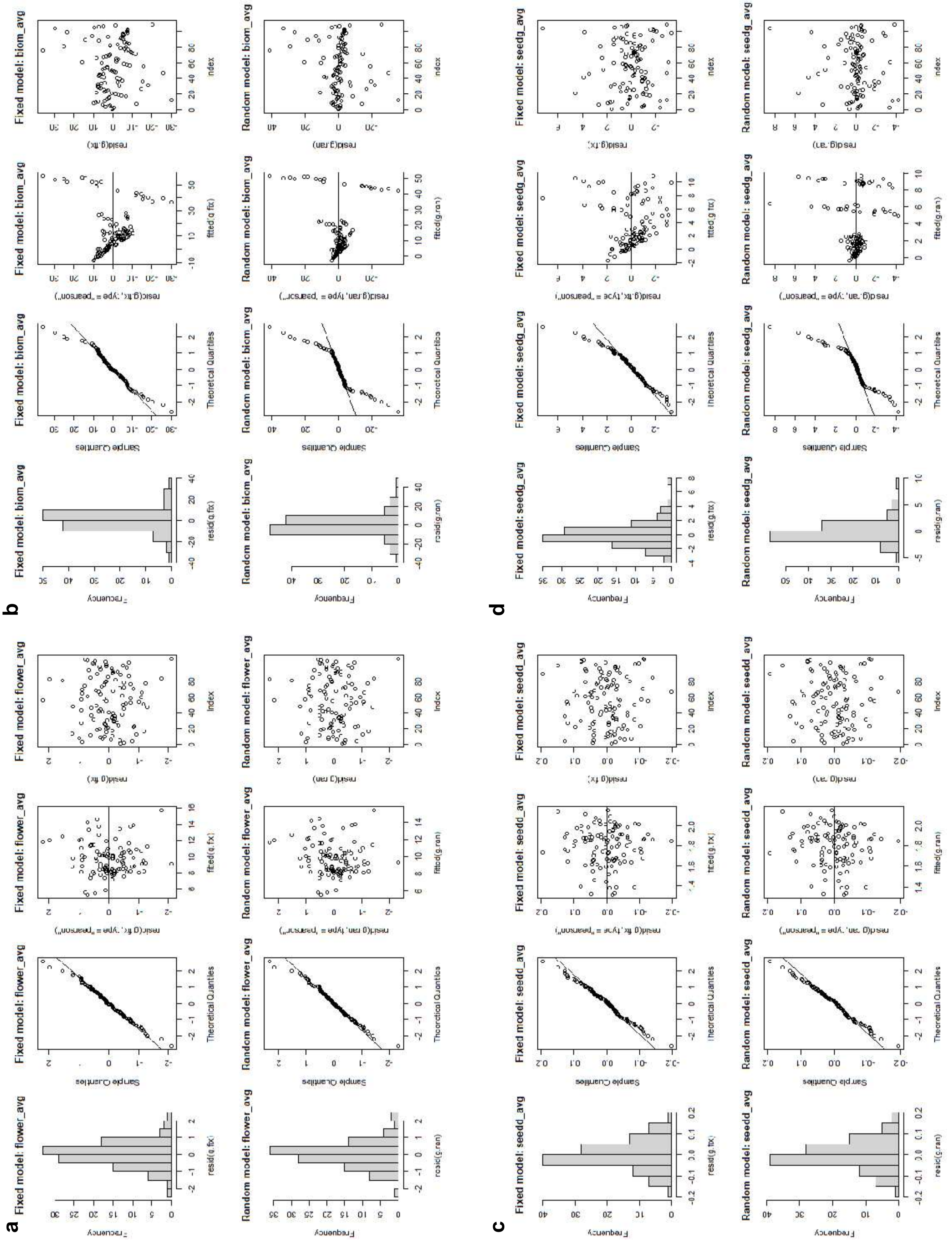

Figure S5: Residual plots for fixed and random effect models. For each trait, residuals for fixed (upper) and random (lower) model are reported. From the left, the distribution of residuals, the quantile-quantile plot, fitted values plot, index plot **a.** flowering time. **b.** dry biomass. **c.** seed diameter **d.** seed weight per plant.

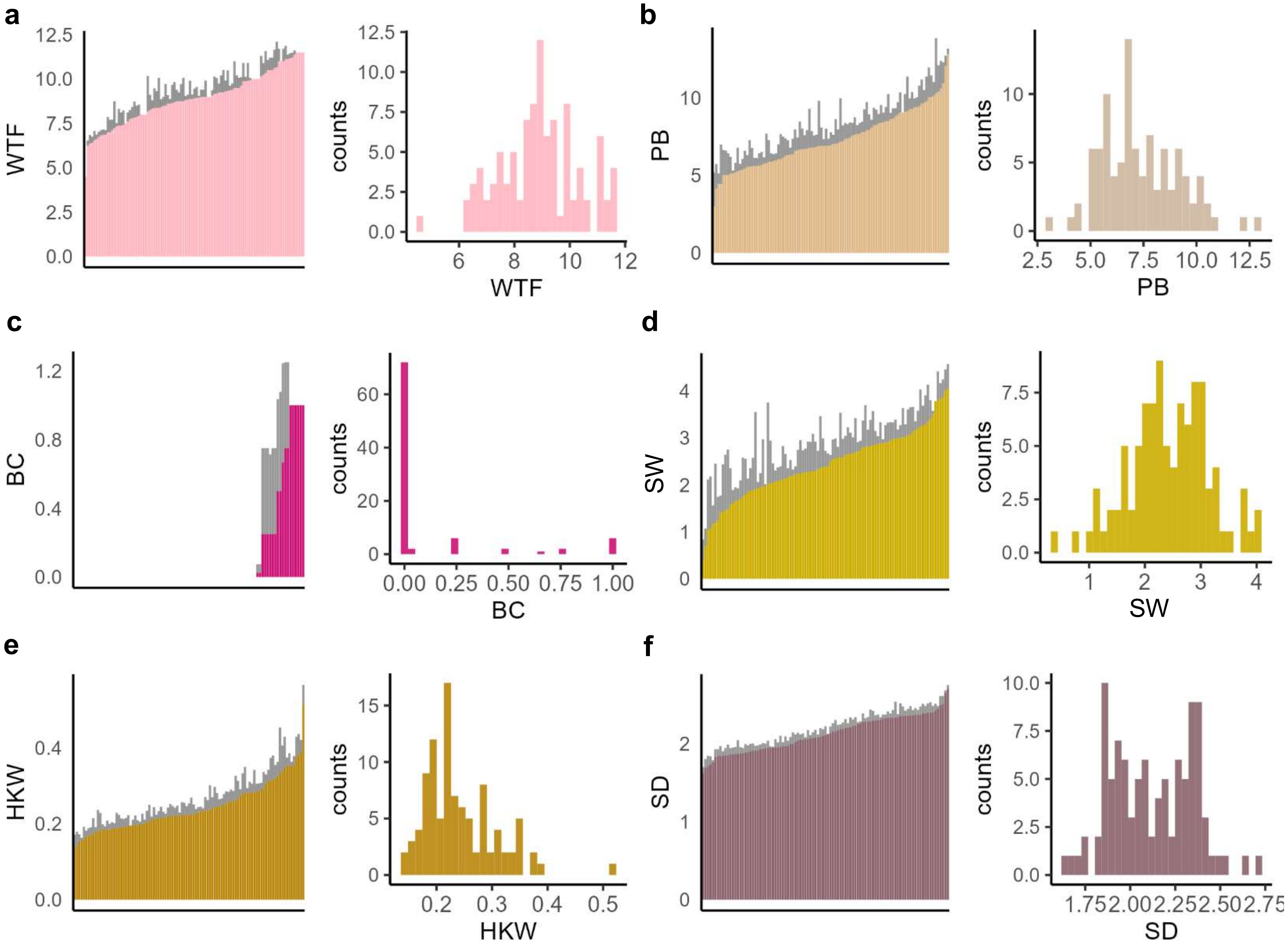

Figure S6: Distributions of traits scored in a quinoa diversity panel. For each scored trait, a barplot and histogram are shown, to illustrate the distribution of the phenotype. The x-axis of each barplot represents all scored accessions, sorted by the phenotype scores. **a.** Weeks-to-flowering (WTF), **b.** Plant dry biomass after harvest (PB) expressed in g, **c.** Stem betalain content (BC) scored between 1-5 for individual replicates and normalized on a scale from 0 to 1, **d.** total seed weight per plant expressed in grams weight of total seeds (SW), **e.** Hundred-kernel-weight (HKW) expressed in g, **f.** Seed diameter (SD) expressed in mm.

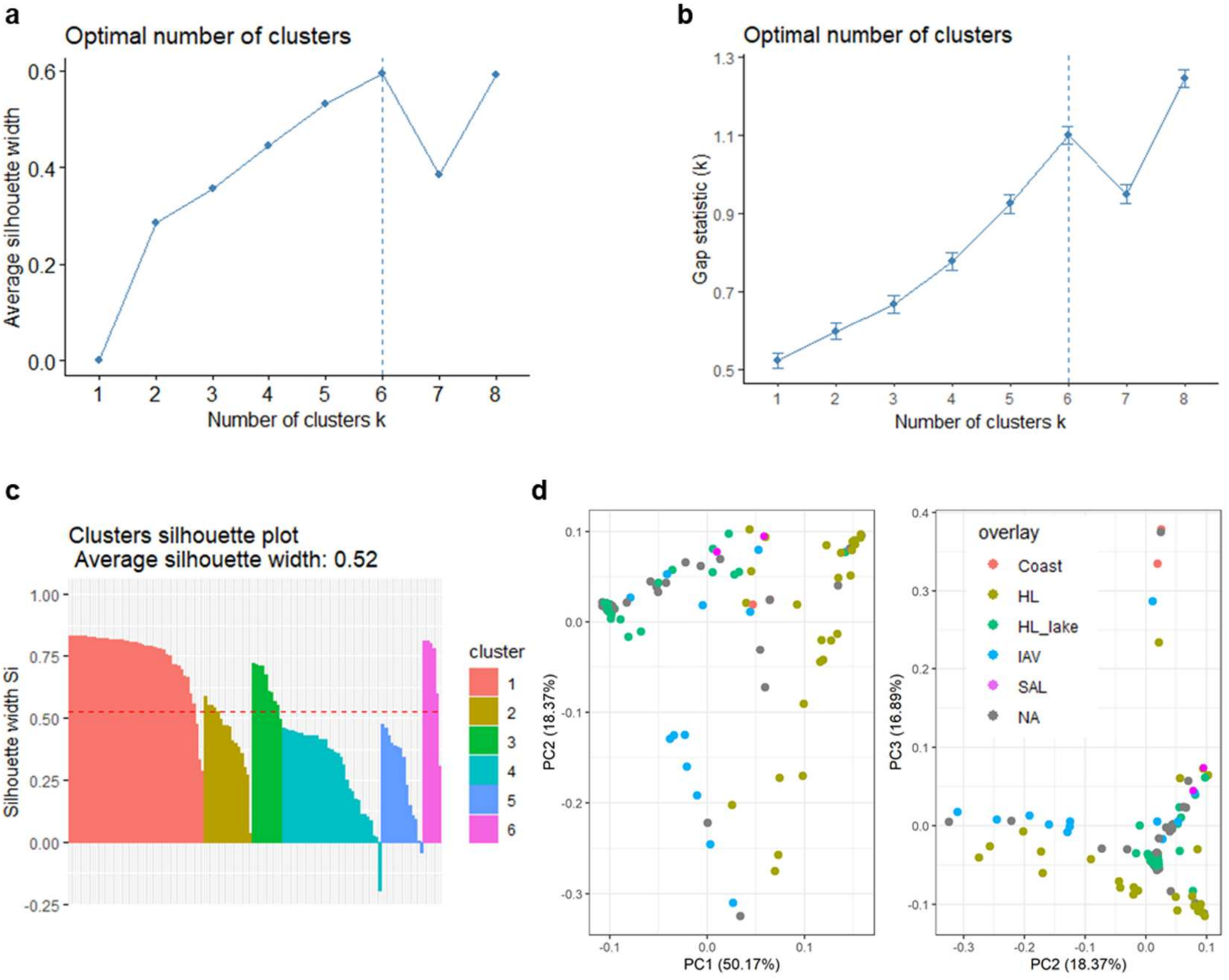

Figure S7: k-means clustering of population structure. a. Optimal number of cluster computed using average silhouette width. **b.** Optimal number of clusters computed using gap statistic **c.** Plot illustrating average Silhouette width across all clusters. **d.** Genotype-based population structure (PC12-PC23), overlayed with ecotype annotations based on the IPK metadata and our knowledge on germplasm origin. Coast: coastal, HL: highland, HL_lake: highland area around lake Titicaca, IAV: inter-andean vallleys, SAL: salar salt flats, NA: no annotation.

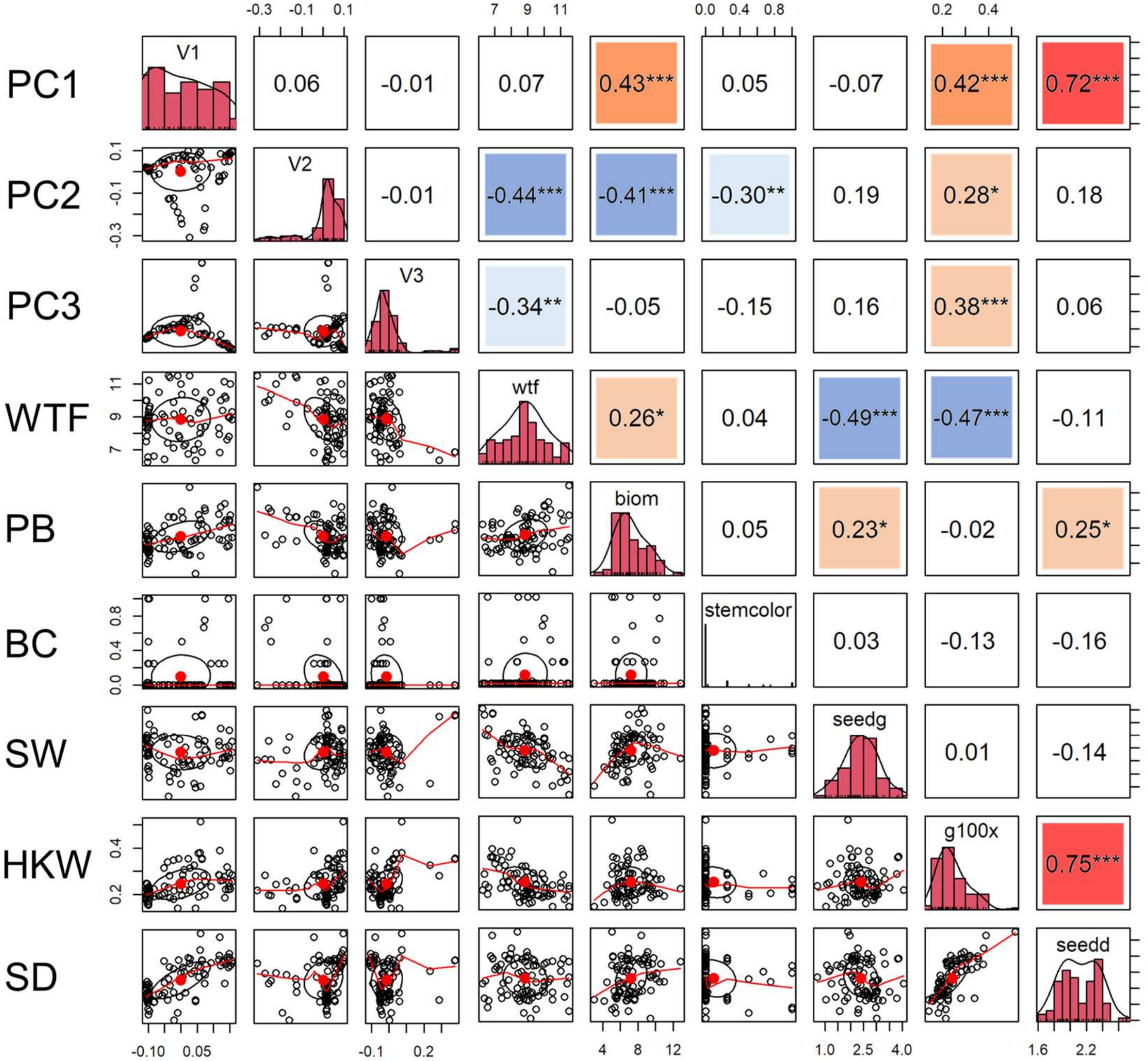

Figure S8: Correlations between scored traits, including population structure. Trait correlations are plotted. The first three components from population structure are included, to investigate subpopulation-related trends. Significant Pearson coefficients are highlighted in colors (red > 0.7, dark orange > 0.4, light orange > 0.2, light blue < -0.2, medium blue < -0.4.

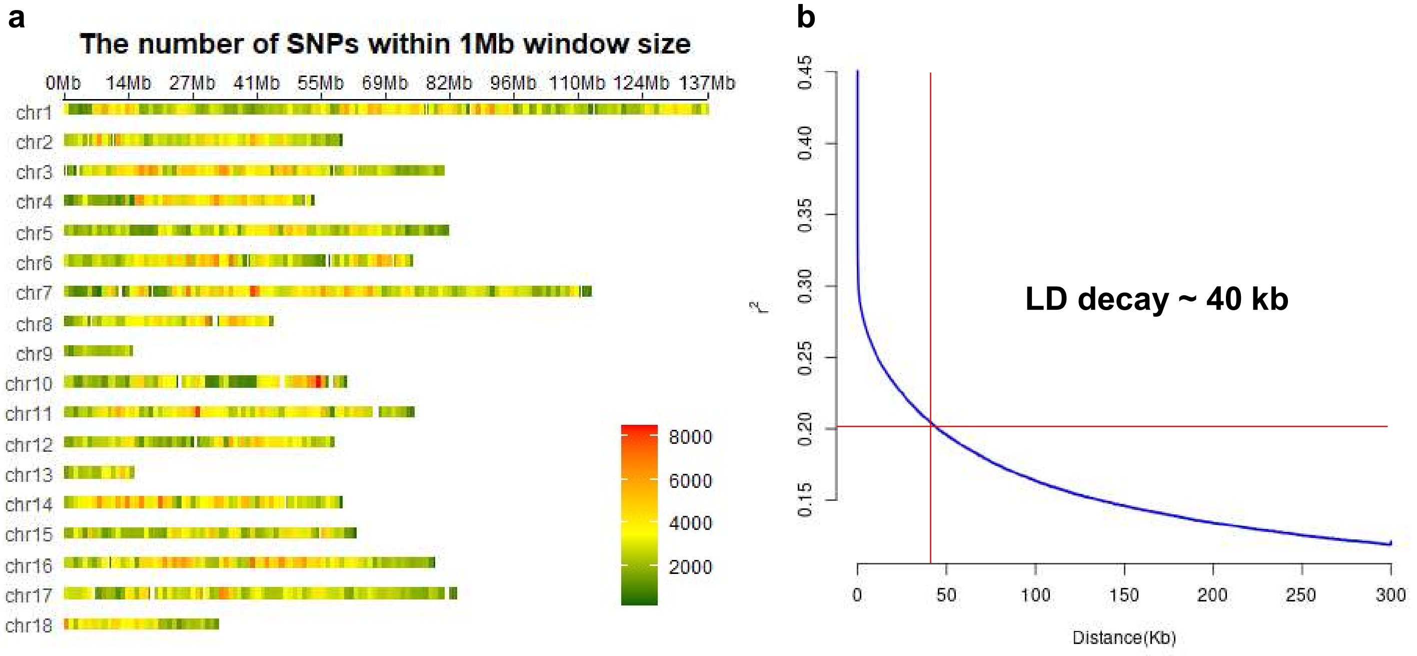

Figure S9: SNP density and linkage disequilibrium decay. a. For each chromosome, the number of SNPs within single 1Mb regions is summarized in color form according to the scale. **b.** LD decay plot calculated across all accessions. Red lines intersect where linkage coefficient r2 decays below 0.2.

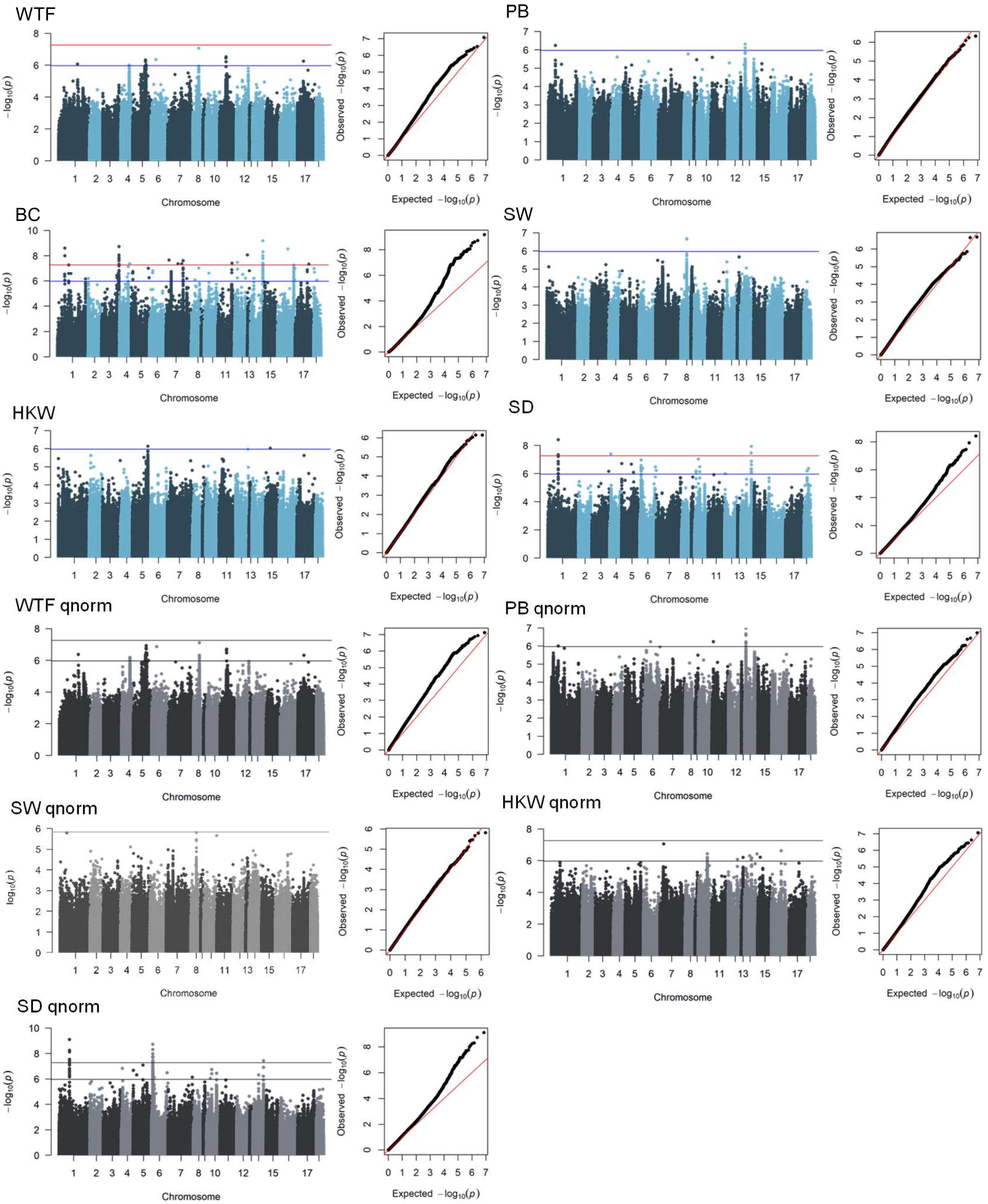

Figure S10: GWAS analysis, GEMMA output for of all traits. Analyses for quantitative traits were carried out using both raw phenotype data (blue manhattan plots) and quantile-normalized phenotype (grey manhattan plots). Weeks to flowering (WTF), dry biomass post-harvest (PB), stem pigmentation due to betalain content (BC), total seed weight per plant (SW), hundred-kernel-weight (HKW), seed diameter (SD). For BC, only raw data were used. For each analysis, Manhattan plot and quantile-quantile plot are shown. Blue line: suggestive threshold, red line: Bonferroni threshold

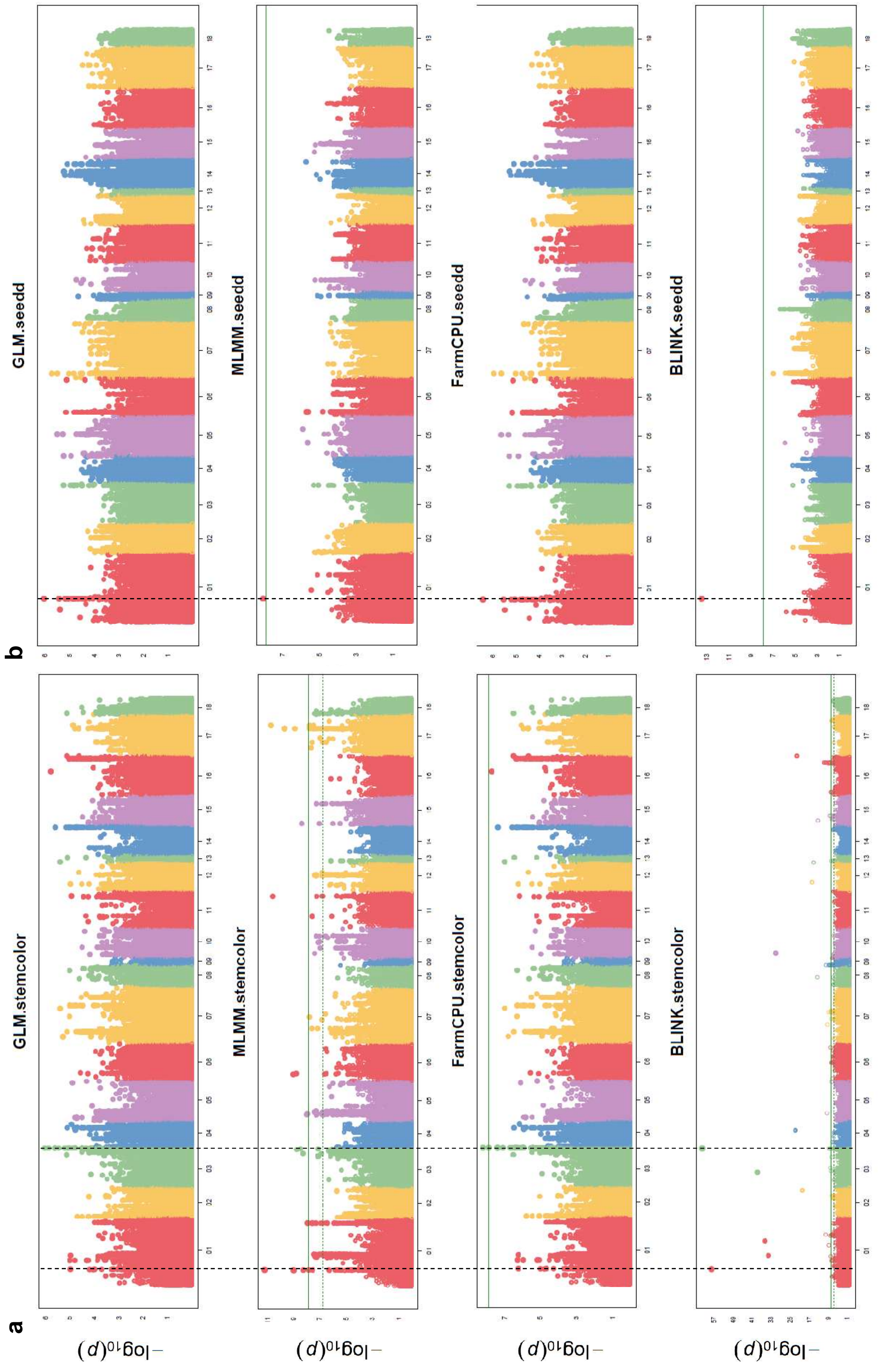

Figure S11: GWAS analysis, GAPIT methods output. Analysis results obtained using GLM, MLMM, FarmCPU and BLINK methods are shown in mahattan plots. Green horizontal lines denotes Bonferroni significance threshold. Green dashed lines denote False Discovery Rate (FDR) threshold. Where one or more methods identify a significant peak previously found with GEMMA LMM, vertical dark dashed lines are drawn. Results are shown for **a.** Stem betalain content and **b.** Seed diameter.

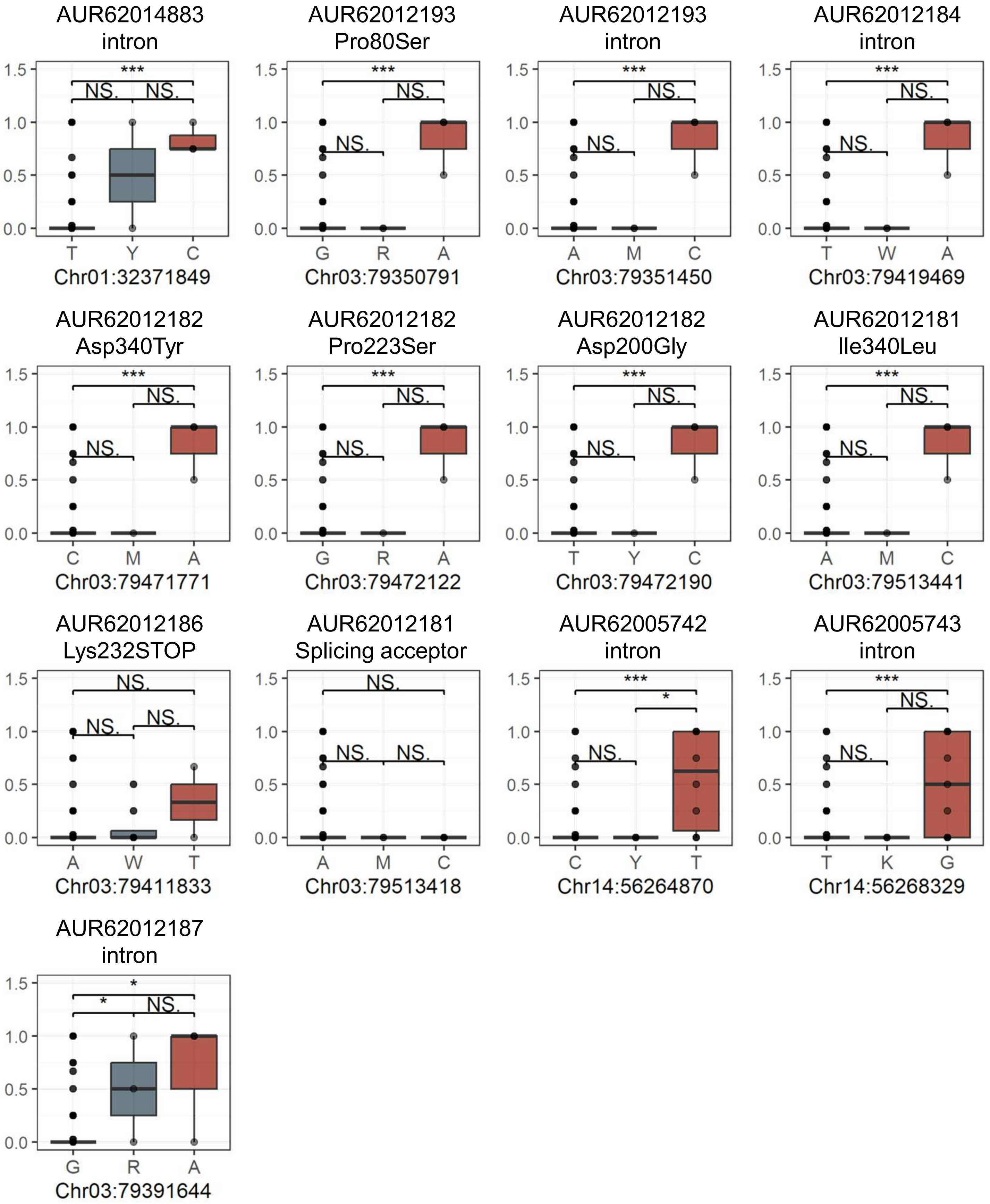

Figure S12: SNPs phenotype distributions, stem pigmentation. Boxplots representing phenotype distributions for chosen SNPs within genes found in significant loci. NS: non-significant, * $p < 0.05$, *** $p < 0.001$ (Mann-Whitney non-parametric test). Heterozygous genotypes are expressed in single letter code.

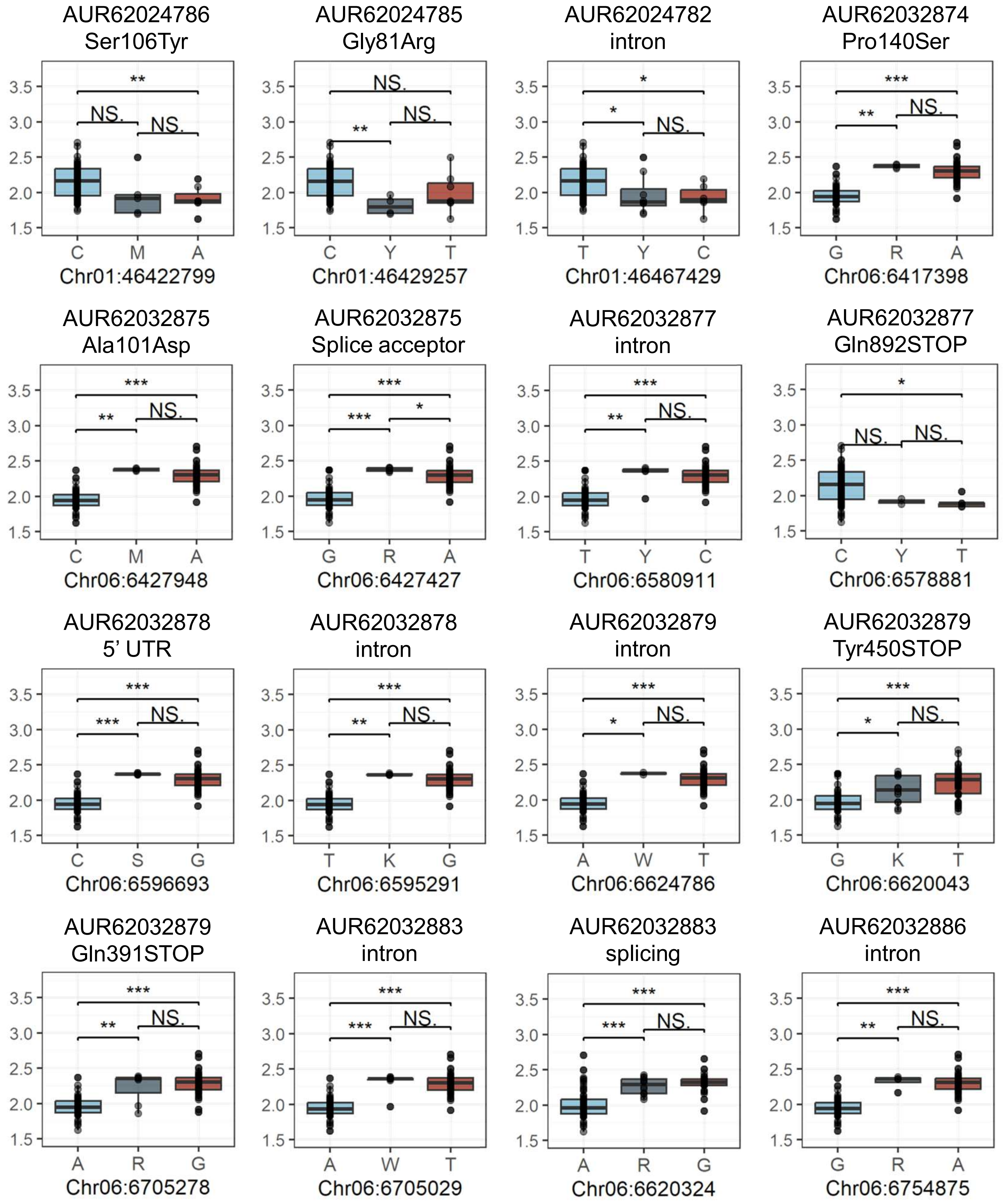

Figure S13: SNPs phenotype distributions, seed diameter. Boxplots representing phenotype distributions for chosen SNPs within genes found in significant loci. NS: non-significant, * $p < 0.05$, *** $p < 0.001$ (Mann-Whitney non-parametric test). Heterozygous genotypes are expressed in single letter code.
